# The protective effects of hyperoside on Ang II-mediated apoptosis of bEnd.3 cells and injury of blood-brain barrier model in vitro

**DOI:** 10.1186/s12906-022-03635-9

**Published:** 2022-06-13

**Authors:** Yu Yan Xie, Yun Wei Lu, Gu Ran Yu

**Affiliations:** 1 Department of Neurology, Jiangsu Province Hospital of Chinese Medicine, The Affiliated Hospital of Nanjing University of Chinese Medicine, Nanjing, Jiangsu Province China; 2 Department of Neurology, Xuzhou City Hospital of Traditional Chinese Medicine, Xuzhou, Jiangsu Province China

**Keywords:** Hyperoside, Ang II, Blood-brain barrier, Tight junction, Transcytosis, Cerebral small vessel disease

## Abstract

**Background:**

Hypertension and its associated dysfunction of the blood-brain barrier (BBB) are considered to contribute to cerebral small vessel disease (cSVD). Angiotensin II (Ang II), as an important vasoactive peptide of the renin-angiotensin system (RAS), is not only a pivotal molecular signal in hypertension, but also causes BBB leakage, cSVD and its related cognitive impair. Hyperoside (Hyp), a flavone glycoside, has antioxidant, antiphlogistic and anti-apoptosis effects. In this study, we investigate the protection of Hyp on apoptosis of bEnd.3 cells and BBB disruption in vitro induced by Ang II.

**Methods:**

We used bEnd.3 cells to imitate a BBB monolayer model and explored the protection of Hyp on Ang II-induced BBB leakage. The apoptotic activity was assessed by TUNEL staining and flow cytometry. The expression of apoptosis pathway related proteins, tight junction proteins and transcytosis related proteins were detected by western blot assay. The BBB model permeability was detected through measuring the flux of sodium fluorescein (Na-F).

**Results:**

We found that Hyp can not only effectively inhibit the apoptosis of bEnd.3 induced by Ang II, but also protect the structural soundness and functional integrity of BBB model by affecting the expression levels of junctional adhesion molecule A (JAM-A), Claudin-5, zonula occludens-1 (ZO-1), Caveolin-1 (Cav-1) and major facilitator superfamily domain-containing protein 2a (Mfsd2a).

**Conclusion:**

Hyp might be a potent compound for preventing Ang II-induced BBB disruption.

**Supplementary Information:**

The online version contains supplementary material available at 10.1186/s12906-022-03635-9.

## Background

Cerebral small vessel disease (cSVD) refers to a series of pathological, imaging and clinical syndromes caused by various reasons affecting cerebral arterioles, microarterioles, venules, microvenules and capillaries. Cognitive and motor disorders are the main clinical manifestations [1, 2]. The causes of cSVD include arteriosclerosis, sporadic or hereditary amyloid angiopathy, inflammatory or immune-mediated vasculitis and other inherited vascular diseases. Hypertension and it caused cerebral arteriosclerosis are the most common cause of sporadic cSVD [3]. The disruption of blood-brain barrier (BBB) caused by hypertension are the main pathological mechanisms [4] in the occurrence and development of cSVD. Angiotensin II (Ang II) is the primary effector peptide of renin-angiotensin system (RAS) [5] which plays a very important role in hypertension. It has been reported that Ang II can promote BBB disruption [6, 7] through several ways such as the direct damage of cerebral vascular endothelial cells (ECs) and effects on substance transportation.

BBB, a dynamic physical barrier which provides a stable microenvironment of central nervous system (CNS) for neural function, comprises of several cellular components including astrocytes, pericytes and ECs [8]. The ECs collaborate with pericytes and astrocytes, controlling the transportation of substances between the brain and blood through paracellular and transcellular pathways. The paracellular diffusion of polar molecules and macromolecules into the CNS are restricted by the tight junctions (TJs) between adjacent ECs. For another, the transcellular pathway across ECs involves caveolar-mediated transcytosis, carrier-mediated, receptor-mediated and adsorptive pathway. Among these, caveolar-mediated transcytosis is the primary mechanism of traffic of substances across the BBB [9].

Any injury of ECs will lead to the disruption of BBB and CNS homeostasis disorder. Previous studies showed that Ang II can induce apoptosis of ECs through angiotensin type I and type II receptors, Fas [10, 11], B-cell lymphoma 2 (Bcl-2) [12] and Caspase family [13, 14]. Besides, Ang II may lead to BBB leakage through paracellular pathways.by influencing TJs and through transcellular pathways by affecting the expression levels of transcytosis-related proteins. It has been reported that Ang II can destroy the distribution of ZO-1 and human vascular endothelial cadherin complex (VE-Cad) on the cell membrane, decrease the levels of JAM-A and Mfsd2a, and increase the expression of Cav-1 [15].

Moreover, Ang II is a pro-inflammatory molecule [16, 17] and it can activate presynaptic angiotensin II type 1 receptors (AT1R) [18, 19]. AT1R activation and vascular inflammation increase the permeability of BBB, induce the leakage of Ang II into the paraventricular nucleus and ventrolateral medulla oblongata, resulting in further excessive activation of the sympathetic excitability [20]. From above we can learn that Ang II exerts an important function in the occurrence and development of cSVD.

Hyperoside (Hyp), a bioactive flavonoid glycoside, is isolated from Herb of Spanishneedles (*Bidens bipinnata* L.). As a traditional Chinese medicine, *Bidens bipinnata* L. has been widely used to treat many diseases in China with the anti-inflammatory, antioxidant and hypolipidemic effects [21–23]. Recent researches have proved that Hyp has comprehensive pharmacological effects such as antioxidant, antiphlogistic, anti-depressant and anti-apoptotic properties [24–27]. Moreover, our previous studies showed that Hyp can protect BBB from Aβ-induced damage [28]. However, its effect on Ang II-induced BBB damage is not fully clear. In this study, we utilized ECs (bEnd.3 cells) to explore the possible mechanism of Hyp on mitigation of BBB hyperpermeability induced by Ang II. In addition, we detected the expression of paracellular and transcellular transport related proteins (JAM-A, Mfsd2a, ZO-1, Claudin-5 and Cav-1) in Ang II-treated ECs to determine the pathway of Hyp protection.

## Materials and methods

### Reagents and antibodies

Hyperoside powder (Cat No. A0090, purity 98.36%) was purchased from Chengdu ManSiTe Biotechnology Co. Ltd. Ang II (Cat No. A9525, purity ≥93%), 3-(4,5-Dimethyl-2-thiazolyl)-2,5-diphenyl-2H-tetrazolium bromide (MTT) (Cat No. M2128), Na-F (Cat No. F6377) and dimethyl sulfoxide (DMSO) (Cat No.67–68-5) were purchased from Sigma. Dulbecco’s modified Eagle’s medium (DMEM) (Cat No. BC-M-005-500 mL), phosphate buffered saline (PBS, PH 7.4) (Cat No. BC-BPBS-01) and fetal bovine serum (FBS) (Cat No. BC-SE-FBS01) were from GIBCO. 0.25% trypsin cell digestive juice (Cat No. C0201), BCA protein concentration determination Kit (Cat No. P0012), one-step TdT-mediated dUTP Nick-End Labeling (TUNEL) Cell apoptosis Detection Kit (Cat No. C1090, Red fluorescence) and DAPI staining solution (Cat No. C1006) were from Beyotime Biotechnology Co. Ltd. Primary Antibodies against Bcl-2-associated X (Bax), Bcl-2, Cytochrome C (Cyt-C), Caspase-3, Caspase-8, Cleaved Caspase-8, Caspase-9 (Cat No. 2772S, 2876S, 4272S, 9662S, 4970S, 8592S, 9508S) were from Cell Signaling Technology, Inc. Zo-1, Claudin-5, JAM-A (Cat No. Ab96587, ab131259, ab180821) were from abcam Company. β-Actin (Cat No. BM0627) were from BOSTER Biological Technology Co. Ltd. Cav-1 (Cat No.16447–1-AP) was from Proteintech. Mfsd2a (Cat No. PA5–21049) was from Thermo Fisher Scientific. The second antibody which were used to bind to the primary antibody and carry a marker HRP that can be detected (Rabbit Antibody and Mouse Antibody) (Cat No. S0001, S0002) were from Affinity Biologicals Co. Ltd. ECL Chemiluminescence solution which was used to detect antibody marked with HRP (Cat No. BL520A) was from Biosharp Company. Annexin V-fluorescein Isothiocyanate (V-FITC)/Propidium Iodide (PI) apoptosis Detection Kit (Cat No. A211–01) was from Vazyme Biotech Co. Ltd.

### Cell culture

The immortalized mouse brain microvascular ECs (bEnd.3 cells) were from ATCC (USA). BEnd.3 cells were cultured in a complete culture medium which contained 10% fetal bovine serum, streptomycin 0.1 mg/mL and penicillin 100 U/mL (Gibco, USA) in an incubator (37 °C, 5% CO_2_/95% air). The culture medium was changed every 2 days. The cells were passaged when they grew to 90%, and the logarithmic growth phase cells were selected for experiment.

### Experimental groups

According to research, Ang II of 0.1 μM can induce apoptosis after 24 h treatment [29]. Therefore, we chose 0.1 μM for 24 h as the experimental concentration and time of Ang II. Confluent bEnd.3 cells were assigned to five groups: control group, Ang II group, Ang II + 100 μM Hyp group, Ang II + 200 μM Hyp group, Ang II + 500 μM Hyp group. Unless specified, otherwise, bEnd.3 cells were reaped for more analyses after incubation for 24 h. For groups treated with both Ang II and Hyp, Hyp was added to bEnd.3 cells 2 h prior to Ang II.

### MTT assay

We used MTT to determine the safe concentrations of Hyp on bEnd.3 cells and the optimal concentrations of Hyp to protect bEnd.3 cells from oxidative stress injury.

1. The logarithmic growth phase cells were inoculated into 96-well plates with a cell number of 1 × 10^4^ per well. The cells were assigned to one control group and 8 model groups (Hyp 10, 20, 50, 100, 200, 500, 800 and 1000 μM) with five accessory wells and one zero-adjusting well in each group. After 24 h of culture, the medium was discarded, 20 μL of MTT (5 mg/mL) was added into each well and incubated for 4 h under 5% CO_2_ and 37 °C. Then the MTT solution was discarded, 150 μL of DMSO was added into each well and shaken for 10 min to solubilize the formazan formed in the viable cells. The absorbance of each well was measured by a Microplate Reader (Bio-Tek, Winooski, VT, USA) at 490 nm. The cell viability = (experimental group value - zeroing group value) / (control group value - zeroing group value) × 100%.
2. It has been reported that Ang II can induce apoptosis by oxidative stress injury [30] so we used H_2_O_2_ as an oxidative stress. The cells were assigned one control group and 8 model groups (H_2_O_2_ 600 μM + Hyp 0, 10, 20, 50, 100, 200, 500 and 800 μM). Each group had five accessory wells plus one zeroing well. After pretreatment with different concentrations of Hyp for 2 h, H_2_O_2_ was added. After 4 h of culture, the absorbance of each well was measured as above. The cell viability was calculated and the optimum administration concentration was determined.

### TUNEL assay for apoptosis

The bEnd.3 cells were cultured on coverslips in 12-well plates. After different treatment according to above 4.3, cells were washed by ice-cold PBS, fixed with 4% paraformaldehyde for 10 min and permeabilized with 0.3% Triton X-100 for 30 min at room temperature. Then the cells were incubated for 1 h with the TUNEL reaction mixture at 37 °C in the dark. After that the nuclei were stained by DAPI for 10 min in the dark at room temperature. Images were taken by fluorescence microscope (Nikon NT-U positive fluorescence microscope, Japan).

### Flow cytometric analysis for apoptosis

The apoptotic rate in bEnd.3 cells was detected by using a Annexin V-FITC/PI Apoptosis Detection Kit. After different treatments, cells were reaped and washed by PBS two times, resuspended in 100 mL 1 × binding buffer. After that, 5 μL of Annexin V-FITC and 5 μL of PI were added into the cell suspension. The cell suspension was incubated in the dark at room temperature for 10 min and then added 400 μL 1 × binding buffer. Finally, apoptotic rate of cells with different treatments were quantified immediately by flow cytometry (FACS Celesta, BD Biosciences), and the data were analyzed by Flow Jo. The cells non-stained with both PI and Annexin V-FITC were thought to be alive cells and the cells stained with PI were regarded as dead cells. The cells stained with Annexin V-FITC were thought to be in early apoptotic stage and both PI and Annexin V-FITC-stained cells were thought to be in late apoptotic stage. The number of early apoptotic cells plus late apoptotic cells is the total number of apoptotic cells.

### Western blot analysis

The cells were inoculated in 6-well plate at the density of 1 × 10^5^ cells/mL. When grew to 80%, they were treated according to above 4.3. Then the cells were harvested after incubation for 24 h, washed by ice-cold PBS 3 times, lysed in radioimmunoprecipitation assay (RIPA) buffer and repeatedly blown on the ice for 5 min. After that, the lysate was collected, cleaved by ultrasonic for 15 seconds and centrifuged at 4 °C, 12000×g for 20 min. Then the supernatant was taken and added 5 × SDS sample buffer, boiled at 100 °C for 10 min. The total protein concentration was detected by BCA protein assay kit. Same amounts (20-40 μg) of protein were loaded on 8–12% SDS-PAGE gel and transferred to PVDF membranes. Membranes were blocked in 5% non-fat milk and then incubated subsequently with the first antibodies overnight at 4 °C. After that the membranes were exposed to the second antibodies at room temperature for 1 h. The blots were washed by TBST three times and visualized using ECL chemiluminescence solution. Then the blots were exposed using Image Lab Gel Imager System (Bio-Rad, China) to obtain the image. Open the data of the image with Image Lab software, select the lanes and the band location to be quantitatively analyzed and remove the background value, click the Analysis Table Options, thus the band density was quantified.

### Establishment of BBB model

The BBB model in vitro was established by using Polyester Transwell inserts (24 well, 0.4 μM pore size, 6.5 mm diameter, Corning Costar, USA) coated with rat tail collagen (Corning, 50 μg/mL in PBS) in advance as the culture support. The models were divided into 6 groups: blank group (no cells), control group, model group (Ang II 0.1 μM), low, middle and high concentration groups of Hyp (50, 200, 500 μM). The bEnd.3 cells were inoculated at a density of 50,000 cells/cm^2^ on the upper surface of the membrane with 100 μL culture medium in the top chamber and 600 μL culture medium in the bottom chamber and cultured in a incubator (37 °C, 5% CO_2_/95% air) according to the guidelines for use of Transwell inserts. Change the medium and measure the transendothelial electric resistance value by MERS00002 resistance meter (Millipore company, USA) for detecting the integrity of BBB models every day.

The transendothelial electrical resistance (TEER) value was the summation of the cells and the filter layer (TEERf), so the resistance value of monolayer bEnd.3 cells was: (TEERec) = (TEER - TEERf) × S (membrane area) (Ω•cm^2^). The S is 0.33cm^2^. When the TEERec reached a stable maximum value and the liquid level leak test was positive, the test was started.

In primary mouse endothelial cell model, the common TEER value was 100–300 Ω•cm^2^ [31]. In the model co-cultured with bEnd.3 cells and primary astrocytes, when the transcellular monolayer resistance is above 300 Ω•cm^2^, it can better simulate the barrier effect of BBB in vivo [32]. However, monolayer cell models usually show lower TEER values. In our experiment, on the 15th–20th day of culture, the resistance across bEnd.3 cells reached a stable maximum value of 34.78 ± 1.77 Ω•cm^2^ and the liquid level leak test was positive.

### Measurement of the permeability of BBB models

The BBB model permeability was detected through measuring the flux of Na-F. After pretreated with or without Hyp for 4 h, 0.1 μM Ang II was added to the inserts for 12 h. Then, the medium in top chamber was replaced by 200 μL Hanks solution containing Na-F (10 mg/L) and the medium in bottom chamber was replaced by 1000 μL Hanks solution. After 30 min,100 μL solution was absorbed from the bottom chamber and its intensity of fluorescence was detected using multifunctional microplate reader (Synergy HT, BIOTEK, USA), the excitation wave length is 485/20 nm and the emission wave length is 528/20 nm. At the same time, the fluorescence intensities of various concentrations of Na-F (0.004, 0.02, 0.1, 0.5, 1, 5 mg/L) were measured and the standard curve of fluorescence intensity was drawn.

By the standard curve the concentration of the Na-F in bottom chamber was calculated. Then the clearance volume was calculated according to the formula: Vc (μL) = (Cb × Vb) /Ct. Cb is the concentration of the bottom chamber, Vb is the volume of the bottom chamber, and Ct is the initial concentration of the top chamber. By plotting the clearance volume against time, the slope represents the clearance rate (μL/min), which can be shown as Permeability (P) × Surface area (S).

The real permeability of Na-F through monolayer bEend.3 cells in vitro (P_e_) can be calculated by the following formula: 1/P_e_S = 1/P_t_S − 1/P_f_S. P_t_S represents the permeability × surface area in each experimental group, P_f_S represents the permeability × surface area in the group with no cell. S (the membrane area) = 0.33 cm^2^.

### Statistical analysis

All tests were replicated three times independently in triplicate. The data of results were expressed by mean ± standard deviation and analyzed by SPSS22.0 software. All data follow the law of normal distribution. T-test was used to compare the mean of two independent samples and one-way analysis of variance (One-Way ANOVA) was used to compare the mean of multiple samples. For all experiments, the differences between groups were statistically significant at *P* < 0.05.

## Results

### The safe and effective concentrations of Hyp on bEnd.3 cells

**Fig. 1:**
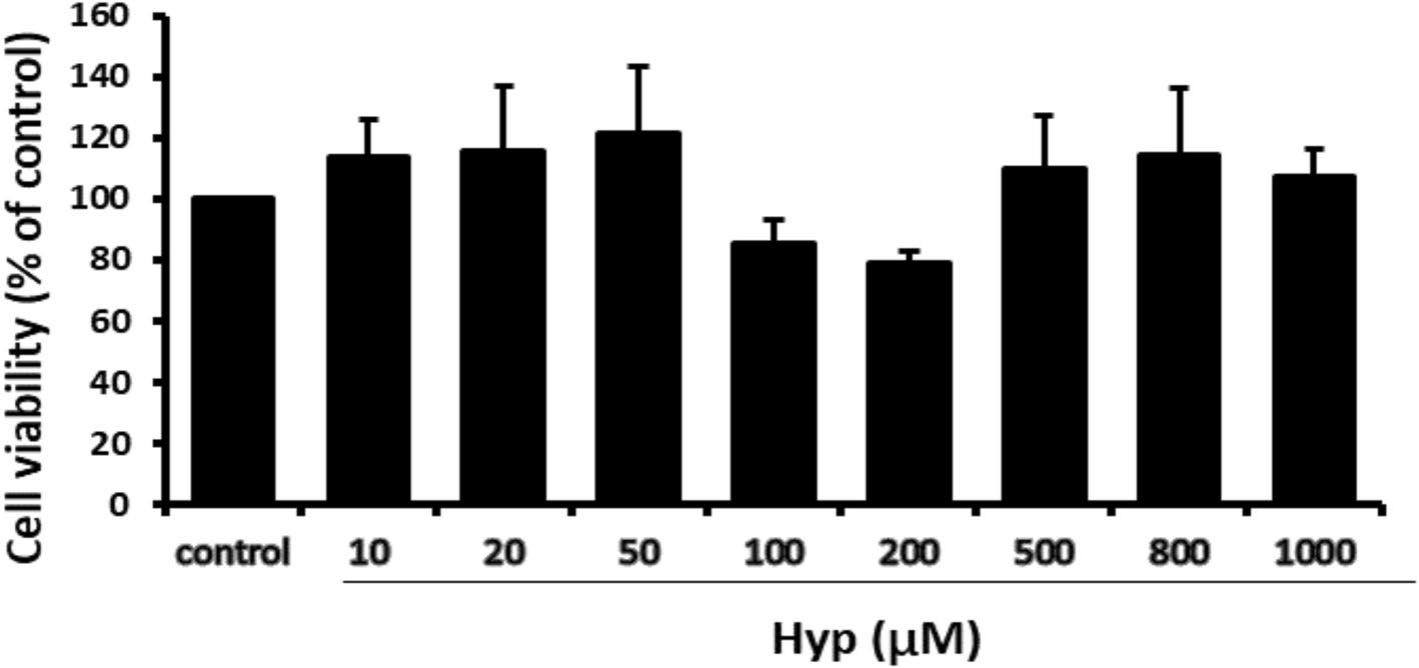
The effects of various concentrations of Hyp on the viability of bEnd.3 cells in vitro. To detect the safety of Hyp, cells were treated with different concentrations of Hyp (10, 20, 50, 100, 200, 500, 800 and 1000 μM) for 24 hr. The cell viability was measured by MTT assay. All the results were represented as mean ± standard deviation of the mean for six replicates from three independent experiments. ^#^*p* < 0.05, ^##^*p* < 0.01 vs control

To explore whether Hyp has toxic effects on bEnd.3 cells, the treatment with various concentrations of Hyp was used. Compared with the control group, there was no statistically significant difference in cell viability among various concentration groups of Hyp. The result suggested that Hyp had no toxic or proliferative effects on bEnd.3 cells (Fig. 1).

**Fig. 2:**
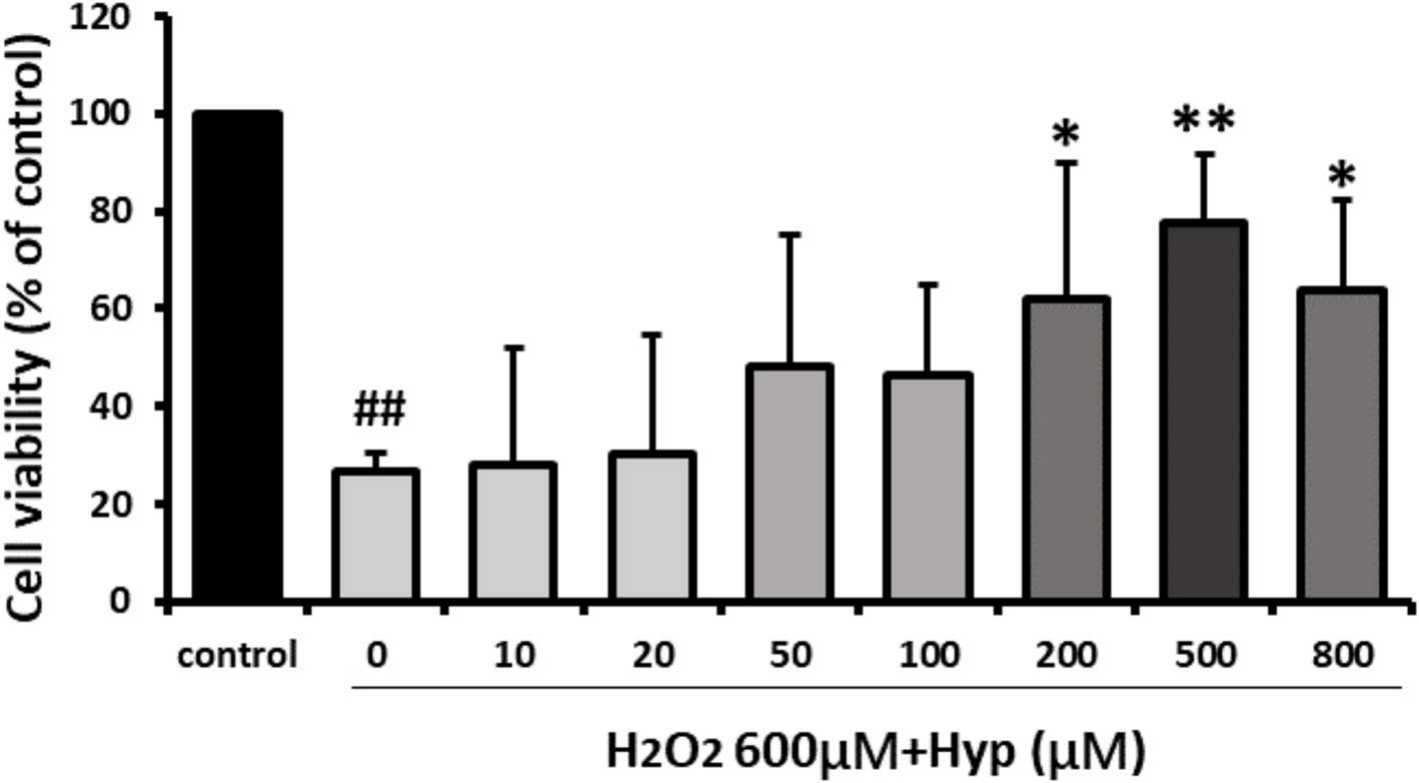
The effects of various concentrations of Hyp against H_2_O_2_-induced damage in bEnd.3 cells in vitro. The cell viability was measured by MTT assay. All the results were represented as mean ± standard deviation of the mean for six replicates from three independent experiments. ^#^*p* < 0.05, ^##^*p* < 0.01 vs control. ^*^p < 0.05, ^**^p < 0.01 vs H_2_O_2_ (600 μM) group

To determine the optimal concentration of Hyp in protecting bEnd.3 cells from oxidative stress injury, bEnd.3 cells were pretreated with different concentrations of Hyp for 2 h, then H_2_O_2_ was added. Compared with the H_2_O_2_-damaged group, 200, 500 and 800 μM Hyp groups had significantly protective effect against H_2_O_2_-induced damage but 500 μM has the strongest protection. In the range of 100, 200, and 500 μM, the protective effect of Hyp increased gradually in a dose-dependent manner (Fig. 2). Therefore, we chose 100, 200 and 500 μM as the low, middle and high dose groups for this study.

### The effect of Hyp on Ang II-induced apoptosis in bEnd.3 cells

**Fig. 3:**
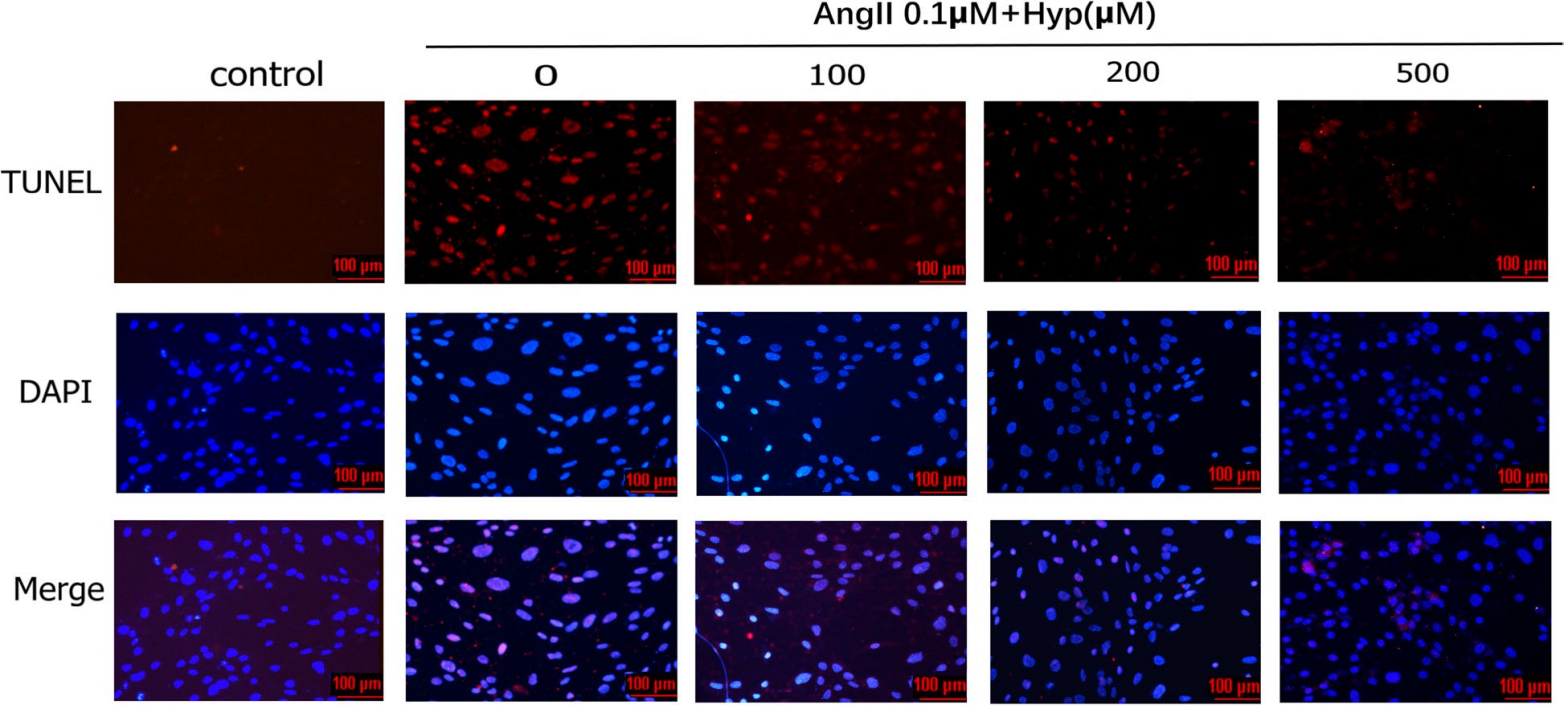
The effects of different concentrations of Hyp on bEnd.3 cells apoptosis under Ang II-treatment in vitro (fluorescence microscopy, 200 × magnification). All nuclei were stained by DAPI with dark blue fluorescence. Apoptotic nuclei stained by TUNEL presented red fluorescence. The experiment was conducted in triplicate

**Fig. 4:**
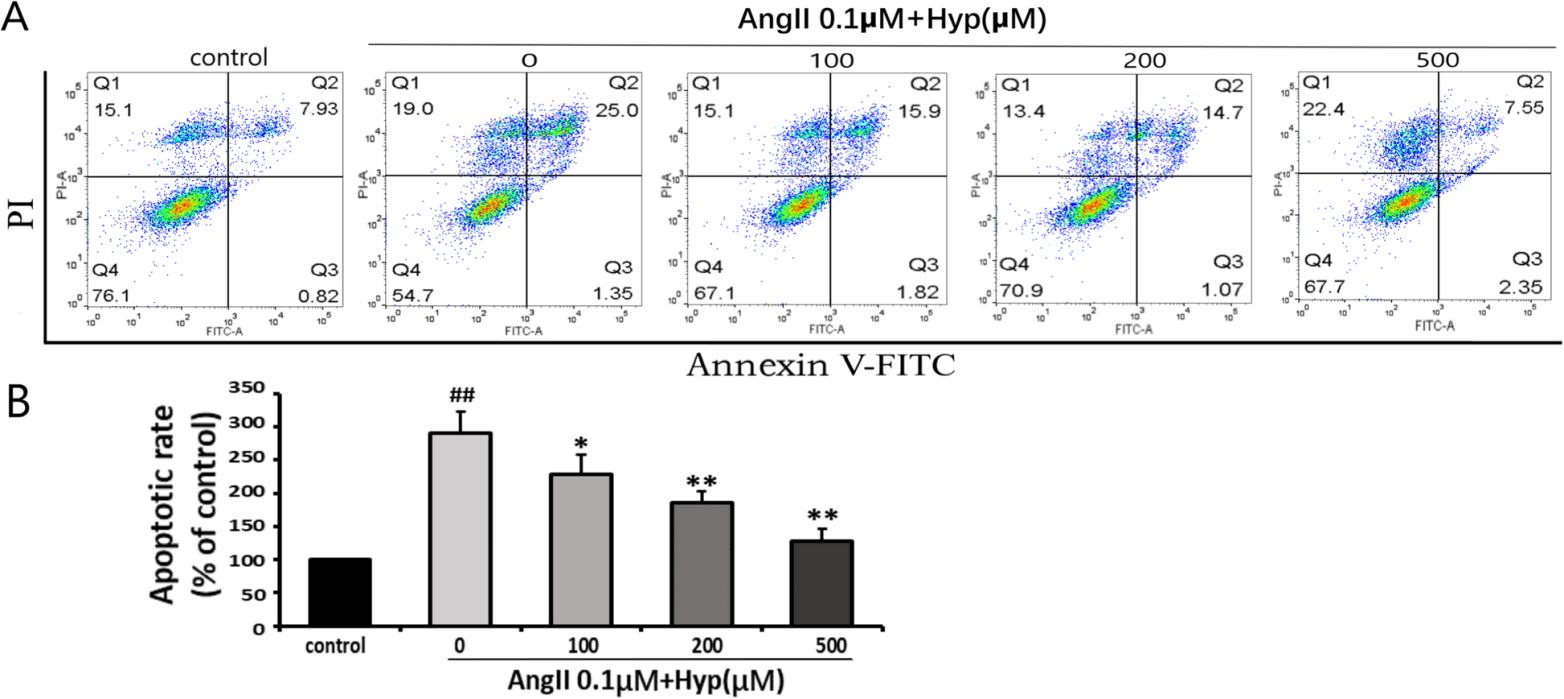
**a** Protection of deferent concentrations of Hyp against Ang II-induced apoptosis in bEnd.3 cells in vitro as detected by flow cytometry (V-FITC/PI) analysis. The data were analyzed by Flow Jo. **b** All the results were represented as mean ± standard deviation of the mean from three independent experiments. ^#^*p* < 0.05, ^##^*p* < 0.01 vs control. ^*^p < 0.05, ^**^p < 0.01 vs Ang II (0.1 μM) group

To determine the function of Hyp on Ang II-induced damage, bEnd.3 cells were pretreated with different concentrations of Hyp for 2 h, then added Ang II. The apoptosis of bEnd.3 cells were observed through TUNEL assay and Flow cytometric analysis. Compared with the model group, the apoptotic rate of cells significantly decreased in low, middle and high dose groups (Figs. 3 and 4). The effects of Hyp were in a concentration-dependent manner.

**Fig. 5:**
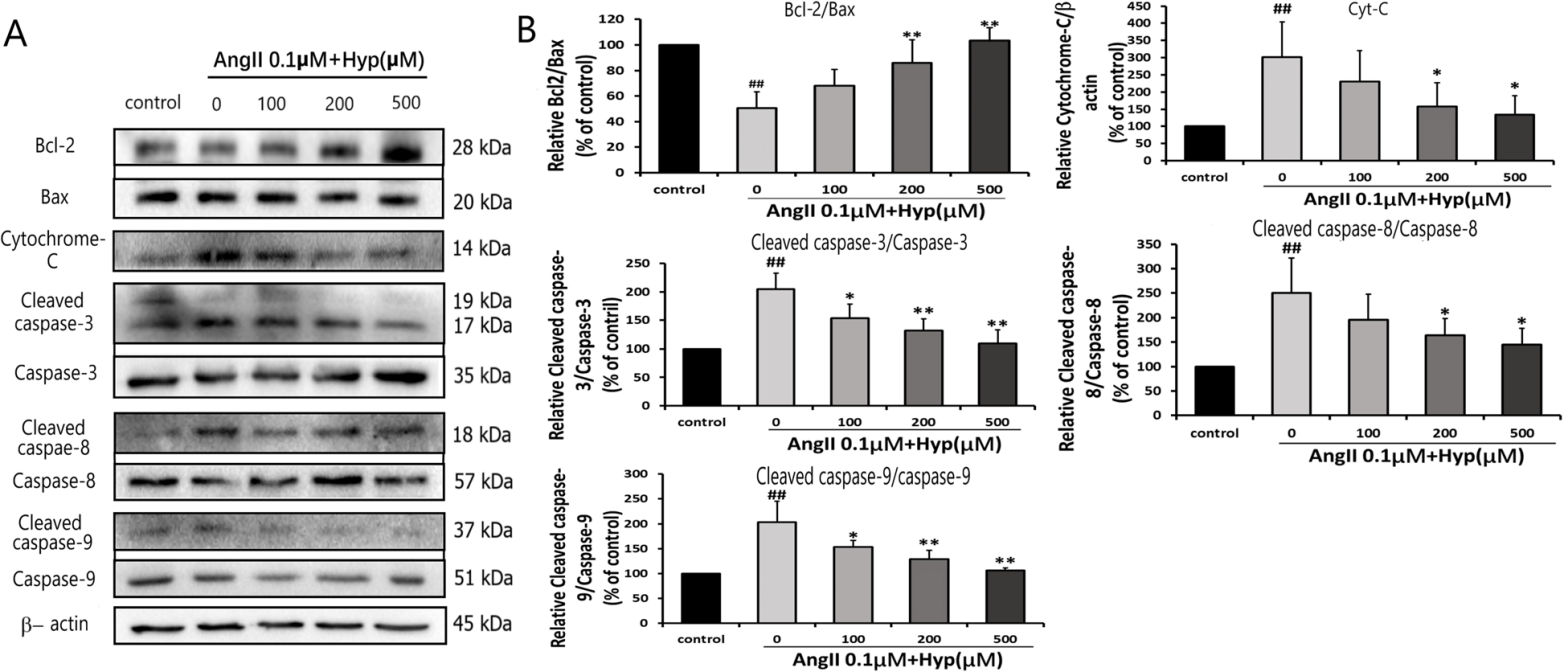
Hyp prevents Ang II-induced apoptosis in bEnd.3 cells via inhibiting mitochondrial and death receptor signaling pathways. **a** Western blot analysis showed the levels of apoptosis-related proteins in five groups. Actin levels were measured for the confirmation of equal amount of protein loading. The blots of Bcl-2 and Bax were cropped from the same gel. The blots of Caspase-3 and Cleaved Caspase-3 were cropped from the same gel. The blots of Caspase-8 and Cleaved Caspase-8 were cropped from the same gel. The blots of Caspase-9 and Cleaved Caspase-9 were cropped from the same gel. **b** The data were represented as mean ± standard deviation of the mean from three independent experiments. ^#^*p* < 0.05, ^##^*p* < 0 .01 vs control. ^*^p < 0 .05, ^**^p < 0.01 vs Ang II (0.1 μM) group

To investigate the protection pathway of Hyp on Ang II-induced apoptosis, we analyzed the apoptotic related proteins expression by western blot after different treatment. Compared with the control group, the expression and activation of Cyt-C, cleaved Caspase-9/Caspase-9, cleaved Caspase-8/Caspase-8 and cleaved Caspase-3/Caspase-3 in the model group were significantly increased, while the activation of Bcl-2/Bax decreased. Hyp can reverse the Ang II-induced increase of cleaved Caspase-9/Caspase-9, cleaved Caspase-8/Caspase-8, cleaved Caspase-3/Caspase-3 and Cyt-C, furthermore, it can increase the ratio of Bcl-2/Bax in Ang II-treated bEnd.3 cells. The above effects of Hyp were in a concentration-dependent manner (Fig. 5).

### The effect of Hyp on TJs and transcytosis-related proteins

**Fig. 6:**
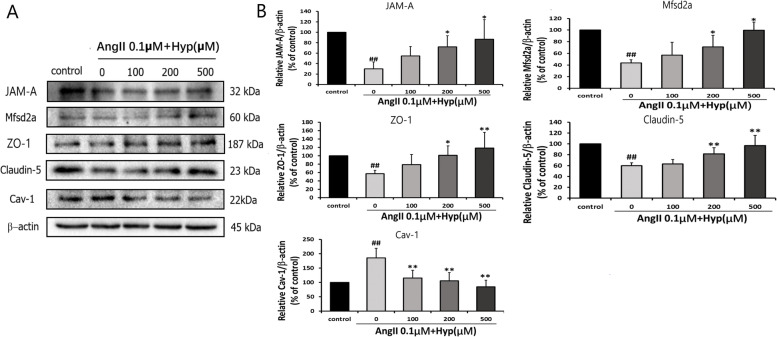
Hyp prevents Ang II-induced damage of BBB via regulating expression of the TJs and transcytosis-related proteins in Ang II-treated bEnd.3 cells. **a** Western blot analysis showed the levels of TJs and transcytosis-related proteins in five groups. Actin levels were measured for the confirmation of equal amount of protein loading. **b** The data were represented as mean ± standard deviation of the mean from three independent experiments. ^#^*p* < 0.05, ^##^*p* < 0.01 vs control. ^*^p < 0.05, ^**^p < 0.01 vs Ang II (0.1 μM) group

Our study showed that 0.1 μM Ang II decreased the levels of JAM-A, Claudin-5 and ZO-1. However, the decreased expression levels of JAM-A, Claudin-5 and ZO-1 were reversed by pretreatment with Hyp. Cav-1 and Mfsd2a are involved in transcytosis of BBB. 0.1 μM Ang II significantly decreased the levels of Mfsd2a and increased that of Cav-1. Hyp reversed the Ang II-induced decrease of Mfsd2a and increase of Cav-1 expression (Fig. 6). The results showed that Hyp could improve the increase of paracellular and transcellular BBB permeability mediated by Ang II through regulation of the levels of JAM-A, Claudin-5, ZO-1, Mfsd2a and Cav-1. The protective effect was in a concentration-dependent way.

### Effect of Hyp on Ang II-mediated BBB injury in vitro

**Fig. 7:**
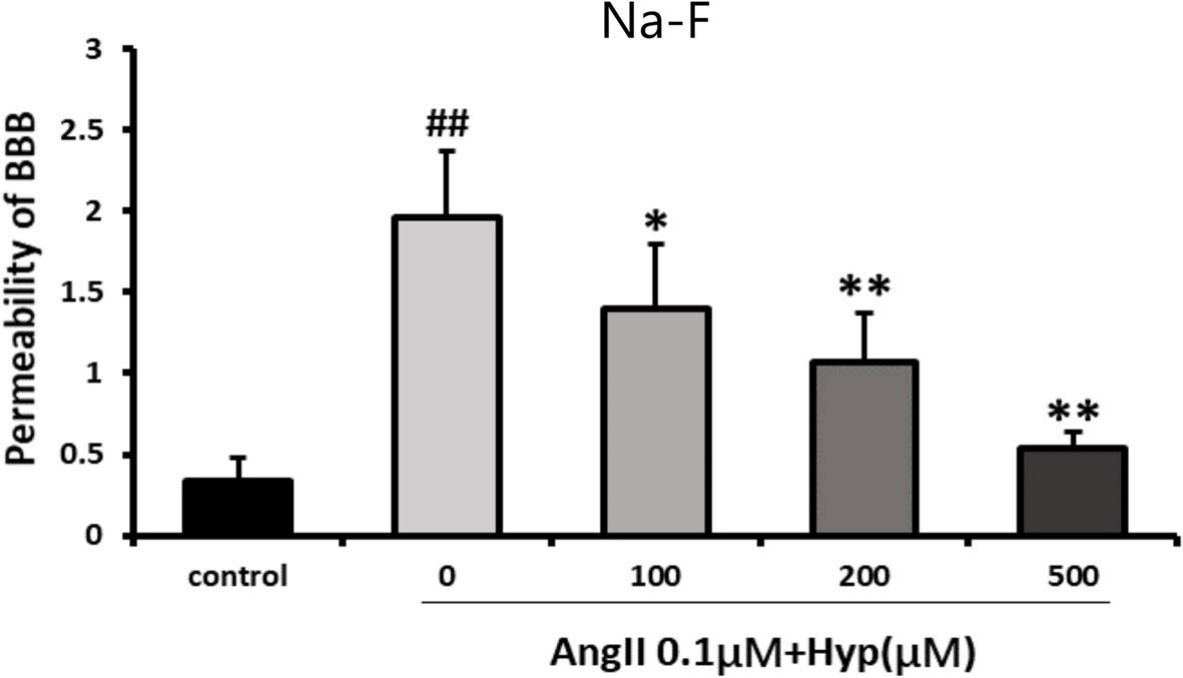
Protection of deferent concentrations of Hyp against Ang II-induced damage of BBB permeability in vitro as detected by determination of Na-F permeability. The data were represented as mean ± standard deviation of the mean from three independent experiments. ^#^*p* < 0.05, ^##^*p* < 0.01 vs control. ^*^p < 0.05, ^**^p < 0.01 vs Ang II (0.1 μM) group

The BBB model permeability was detected through measuring the flux of Na-F. It showed that Ang II increased permeability of BBB, whereas pretreatment with Hyp decreased Ang II-induced permeability damage of BBB. The Hyp protection was in a concentration-dependent way (Fig. 7).

## Discussion

In present study, we established mono-cultured BBB models exposed to Ang II to imitate the BBB damage induced by hypertension. We found that Hyp can relieve BBB leakage induced by Ang II. The potential mechanisms are listed as follows: (a) Hyp protects bEnd.3 cells from Ang II-triggered apoptosis by inhibiting the mitochondrial pathway and the death receptor pathway. (b) Hyp increased the expression of TJs including claudin-5, ZO-1, and JAM-A in Ang II-induced ECs. (c) Hyp inhibited the increase of Ang II-induced transcytosis in BBB by regulating the levels of Cav-1 and Mfsd2a.

We confirmed that Ang II can mediated apoptosis, tight junction protein degradation and abnormal expression of transporters and endocytosis-related proteins. All these will directly lead to BBB dysfunction. On the other hand, BBB dysfunction will further lead to leakage of Ang II into the key brain regions, thus a vicious circle leading to the progression of cSVD occurs. Therefore, protecting the integrity of BBB is the key to the prevention and treatment of cSVD.

Brain ECs are the main components that support the basic structure and function of BBB so the key to protect BBB is to protect ECs. Our study showed that Hyp can effectively protect bEnd.3 cells from oxidative stress injury and Ang II-induced apoptosis. Studies have shown that Ang II may induce apoptosis of endothelial cell by activating Caspase through mitochondria dependent pathway and death receptor pathway [13, 14]. Caspase-8 and Caspase-9 are the initiators of apoptosis, located in the upstream of the cascade. They can activate themselves and downstream Caspase with the participation of other proteins [33, 34]. Caspase-3 is an apoptotic effector, which is located in the downstream of the cascade reaction. Cleaved Caspase-3 acts on specific substrates to cause morphological and biochemical changes, resulting in apoptosis. Caspase-3 is considered to be the key protease of apoptosis [35, 36], once activated, a downstream cascade occurs, which makes apoptosis inevitable. In the death receptor pathway, Caspase-8 is activated by the death receptor on the cell membrane and self-hydrolyzed to form active Caspase-8. Caspase-3 can be directly activated by active Caspase-8 and induce apoptosis [37]. In the mitochondrial pathway, when stimulated by apoptosis stimulators such as DNA damage, cell cycle arrest, toxin and ATP depletion, mitochondrial membrane swells and its permeability increases, resulting in the release of apoptosis-related active substances originally located in the mitochondria, including Cyt-C, apoptosis-inducing factor (AIF) endonuclease [38] and Bcl-2 inhibitor of transcription (Bitl) [39]. Cyt-C can interact with Caspase-9 and apoptotic protease-activating factor-1 (APAF-1) to form aptoptosome. Caspase-9 can self-clip [34], then self-activated Caspase-9 activates Caspase-7, Caspase-6, Caspase-3 and other members in the presence of dATP and ATP, so that apoptosis can continue [40]. Bcl-2 is located outside the mitochondrial membrane and can inhibit the release of Cyt-C by reducing the permeability of the mitochondrial membrane, while Bax increases the mitochondrial membrane permeability and promotes the release of Cyt-C by combining with Bcl-2 [41, 42]. In addition to directly activating Caspase-3, Caspase-8 can also cut the cytoplasmic Bid precursor to form truncated Bid (tBid). The tBid translocates to the mitochondria, triggers the homooligomerization of Bak and Bax, and initiates the release of Cyt-C. Thus, the apoptosis signal is transmitted from the death receptor pathway to the mitochondrial pathway and effectively amplified [34, 43]. The two approaches crisscross and promote each other. Our data suggested that Hyp can exert function of anti-apoptosis through inhibiting the death receptor pathway and mitochondrial pathway.

TJs, including closed proteins (occludin), zonula occludens-1 (ZO-1), claudins and junctional adhesion molecule A (JAM-A), have been proved to play very important role in maintaining BBB function [44]. TJs regulate the diffusion of ions and solutes through the paracellular path and limit the free movements of lipids and proteins from the apical and basolateral cell surfaces [45]. The loss of any one of them may result in seriously damage of BBB. ZO-1 combines the blocking protein with the cytoskeleton system to form a stable tight junction system [46]. Claudin-5 has been identified as a TJ protein highly expressed in BBB and its deletion will cause leakage of small molecules in BBB [47]. JAM-A also exerts an important function in regulating paracellular permeability of endothelial and maintaining the integrity of the BBB [48, 49]. Our data showed that Ang II reduced the expression levels of Claudin-5, ZO-1 and JAM-A in bEnd.3 cells, resulting in an increased permeability of BBB through paracellular pathway, and Hyp can reduce Ang II-induced BBB leakage by up-regulating the expression of these three.

Besides apoptosis of ECs and breakdown of TJs, BBB disruption is also closely associated with enhancement of transcellular pathway via caveolar vesicles. Caveolae is a special vesicular concave structure located on the cell membrane. It mainly transports macromolecular substances in blood and participates in the regulation of ECs transcellular transport [50]. Its surface marker protein Cav-1 plays an important role in maintaining its normal function. Previous studies have confirmed that the upregulation of Cav-1 expression may damage the integrity of BBB. The up-regulated expression of Cav-1 can increase the amount of caveolae, enhance the transcellular transport, and, consequently, the permeability of BBB. The Cav-1-mediated transcellular transport pathway may cooperate with other transport pathways to induce the opening of the BBB [51]. Moreover, Cav-1 can mediate claudin-5 redistribution and damage BBB [52]. P-glycoprotein (P-gp), which plays an important role in brain protection, is closely related to Cav-1. The level of P-gp/Cav-1 interaction can modulate brain endothelial angiogenesis and P-gp dependent cell migration [53]. Cav-1 produces negative feedback regulation on P-gp and jointly maintains the function of BBB [54]. Cav-1 is generally enriched in microenvironment which is rich in cholesterol, saturated phospholipids, sphingomyelin and phosphatidylethanolamine plasmalogen (PE-p). Mfsd2a, a member of the major facilitator superfamily (MFS) of membrane proteins, is specifically expressed in microvascular ECs and responsible for transporting lysophosphatidylcholine (LPC) bound to long-chain fatty acids (LPC-FA) into the brain. It is an important transporter to maintain the function of BBB [55]. Docosahexaenoic acid (DHA), a highly unsaturated fatty acid, can disrupt the ordered domain of Cav-1 and transfer it away from the cell membrane, so that cells cannot form caveolae. DHA is mainly transported into the brain by binding to LPC. In cerebral microvascular ECs, Mfsd2a mediates the absorption of LPC-DHA and induces the microenvironment enriched in DHA and scarce in PE-p, which is not conducive to the assembly of functional caveolin domains and vesicle formation. Thus, Mfsd2a inhibits Cav-1-mediated transcytosis, thereby maintaining the integrality of BBB. In Mfsd2a deficient mice, the number of Cav-1 positive vesicles increased, and so did the transcytosis rate of ECs [56]. Our study showed that Ang II can increase permeability of BBB model by affecting the expression of Cav-1 and Mfsd2a, while Hyp can suppress transcytosis and relieve Ang II-triggered BBB leakage by increasing Mfsd2a expression and decreasing Cav-1 expression.

## Conclusions

In summary, Hyp can not only effectively inhibit the apoptosis of bEnd.3 induced by Ang II, but also protect the structural and functional integrity of BBB in vitro by affecting the expression of Claudin-5, ZO-1, JAM-A, Cav-1 and Mfsd2a. Our research provides a preliminary experimental foundation for Hyp to become a potential drug for effective prevention and treatment of cSVD.

## Supplementary Information

**Additional file 1.**

## Data Availability

All data generated or analysed during this study are included in this manuscript.
