# Supplementary figures and images for: The protective effects of hyperoside on Ang II-mediated apoptosis of bEnd.3 cells and injury of blood-brain barrier model in vitro
Source: BMC Complement Med Ther. 2022 Jun 13;22:157. doi: 10.1186/s12906-022-03635-9 (PMC9195266; doi:10.1186/s12906-022-03635-9)

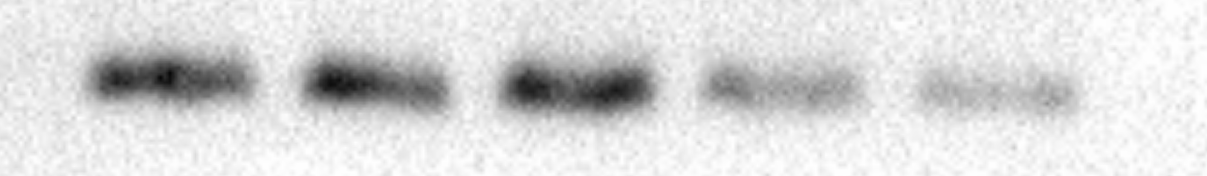

Supplement: Supplementary file 1 — Additional file 1. [file 12906_2022_3635_MOESM1_ESM.zip › 1-actin(Claudin-5).tif]

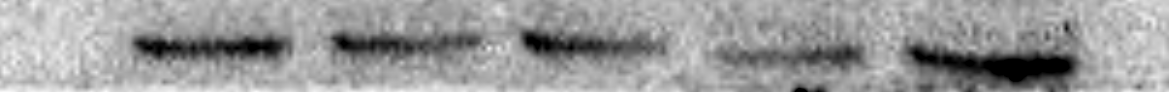

Supplement: Supplementary file 1 — Additional file 1. [file 12906_2022_3635_MOESM1_ESM.zip › 1-actin(Cytochrome-C).tif]

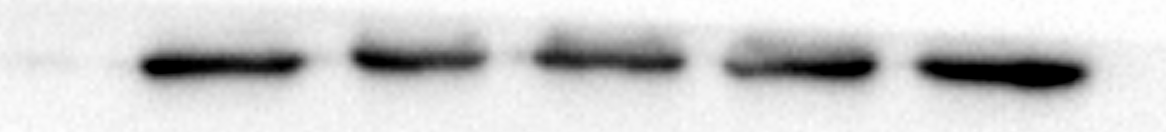

Supplement: Supplementary file 1 — Additional file 1. [file 12906_2022_3635_MOESM1_ESM.zip › 1-Caspase-3.tif]

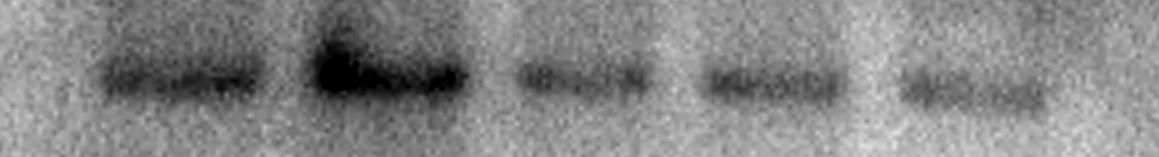

Supplement: Supplementary file 1 — Additional file 1. [file 12906_2022_3635_MOESM1_ESM.zip › 1-Cav-1.tif]

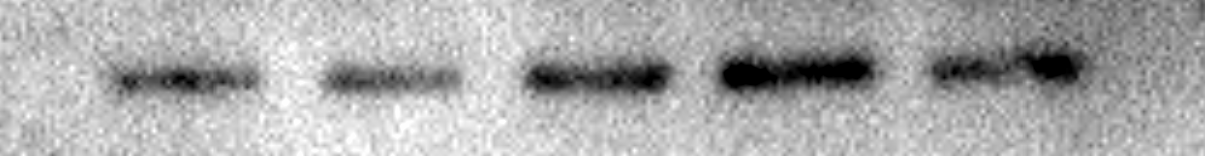

Supplement: Supplementary file 1 — Additional file 1. [file 12906_2022_3635_MOESM1_ESM.zip › 1-Claudin-5.tif]

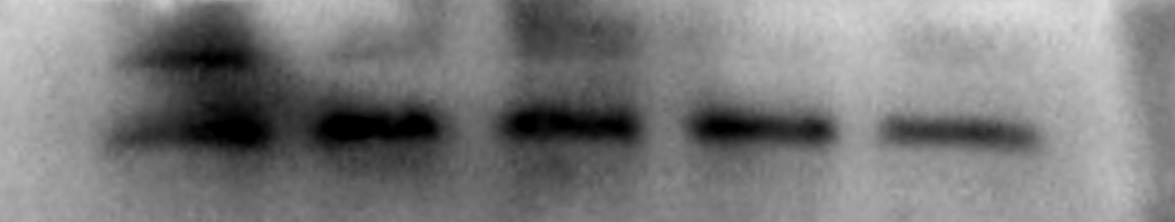

Supplement: Supplementary file 1 — Additional file 1. [file 12906_2022_3635_MOESM1_ESM.zip › 1-Cleaved caspase-3.tif]

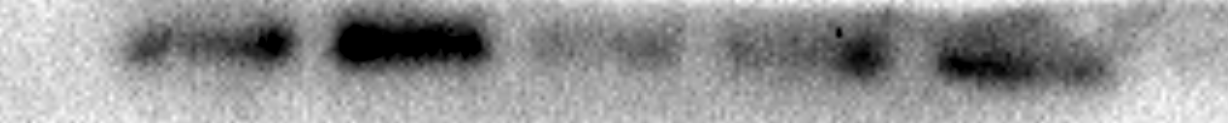

Supplement: Supplementary file 1 — Additional file 1. [file 12906_2022_3635_MOESM1_ESM.zip › 1-Cytochrome-C.tif]

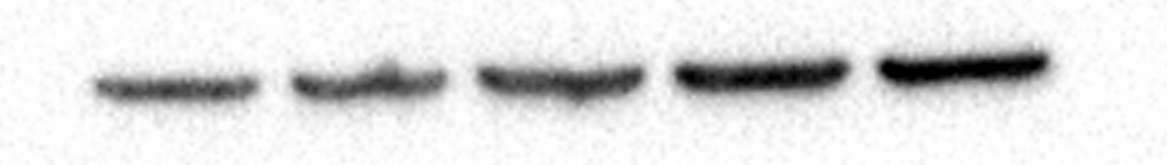

Supplement: Supplementary file 1 — Additional file 1. [file 12906_2022_3635_MOESM1_ESM.zip › 2. actin(Cav-1).tif]

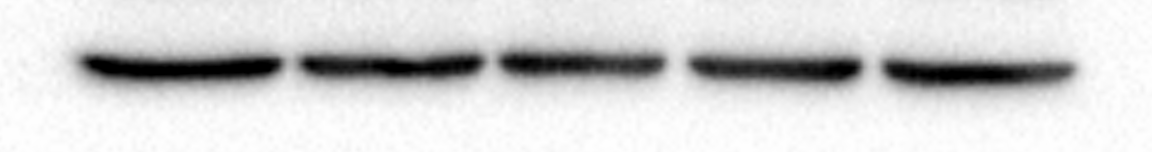

Supplement: Supplementary file 1 — Additional file 1. [file 12906_2022_3635_MOESM1_ESM.zip › 2. actin(Claudin-5).tif]

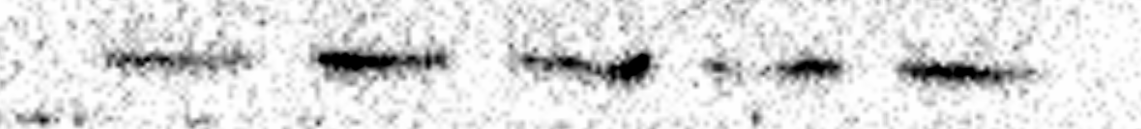

Supplement: Supplementary file 1 — Additional file 1. [file 12906_2022_3635_MOESM1_ESM.zip › 2. actin(Cytochrome-C).tif]

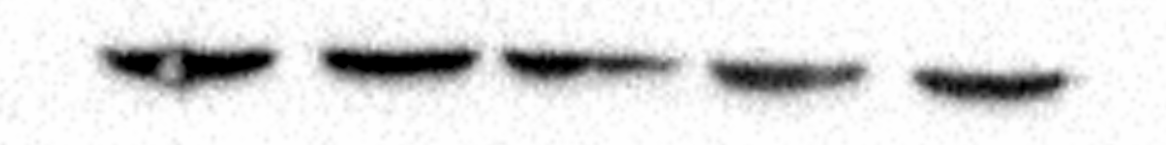

Supplement: Supplementary file 1 — Additional file 1. [file 12906_2022_3635_MOESM1_ESM.zip › 2. actin(JAM-A).tif]

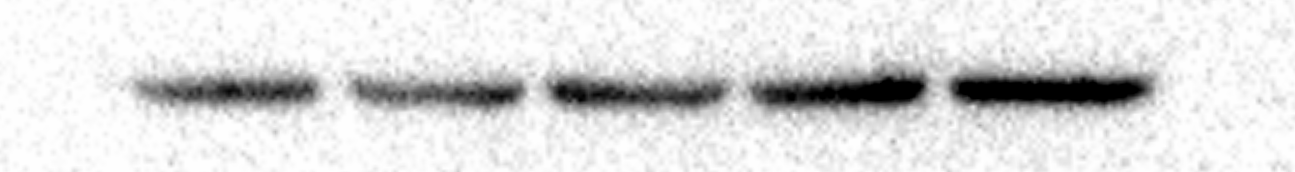

Supplement: Supplementary file 1 — Additional file 1. [file 12906_2022_3635_MOESM1_ESM.zip › 2. actin(ZO-1).tif]

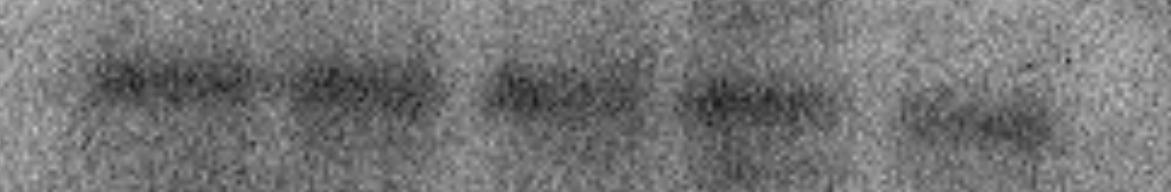

Supplement: Supplementary file 1 — Additional file 1. [file 12906_2022_3635_MOESM1_ESM.zip › 2. Cav-1.tif]

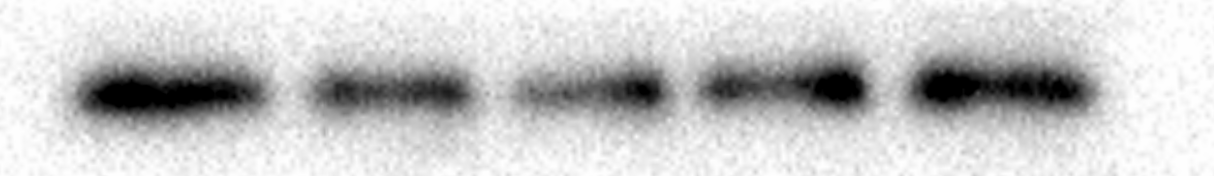

Supplement: Supplementary file 1 — Additional file 1. [file 12906_2022_3635_MOESM1_ESM.zip › 2. Claudin-5.tif]

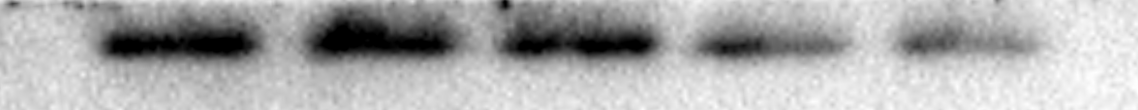

Supplement: Supplementary file 1 — Additional file 1. [file 12906_2022_3635_MOESM1_ESM.zip › 2. Cytochrome-C.tif]

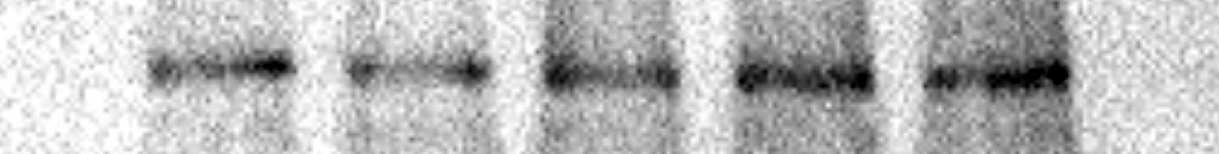

Supplement: Supplementary file 1 — Additional file 1. [file 12906_2022_3635_MOESM1_ESM.zip › 2. ZO-1.tif]

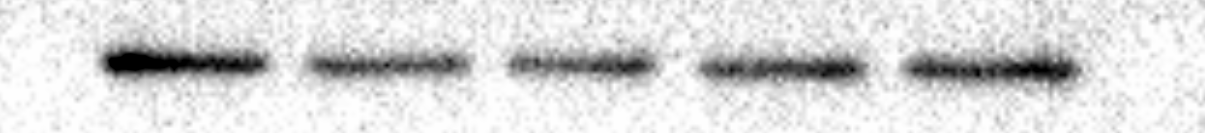

Supplement: Supplementary file 1 — Additional file 1. [file 12906_2022_3635_MOESM1_ESM.zip › 2.Caspase-3.tif]

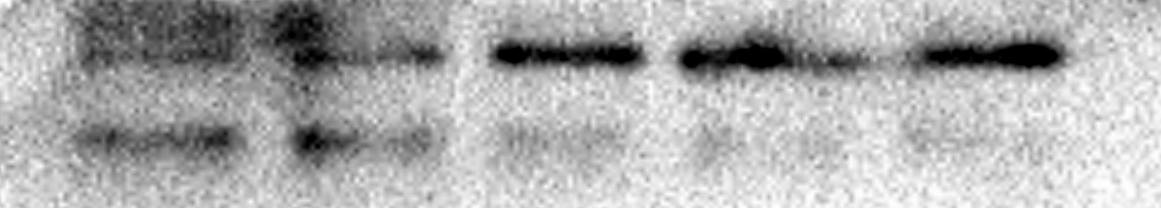

Supplement: Supplementary file 1 — Additional file 1. [file 12906_2022_3635_MOESM1_ESM.zip › 2.Cleaved Caspase-3.tif]

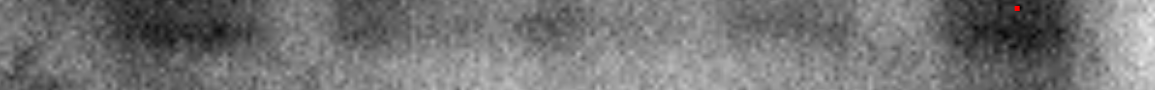

Supplement: Supplementary file 1 — Additional file 1. [file 12906_2022_3635_MOESM1_ESM.zip › 2.JAM-A.tif]

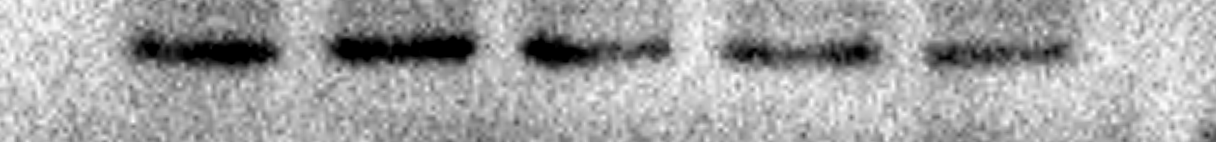

Supplement: Supplementary file 1 — Additional file 1. [file 12906_2022_3635_MOESM1_ESM.zip › 3. Cav-1.tif]

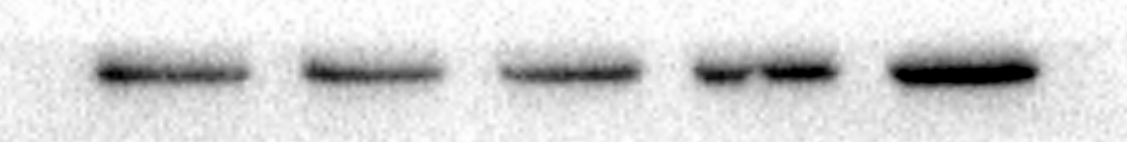

Supplement: Supplementary file 1 — Additional file 1. [file 12906_2022_3635_MOESM1_ESM.zip › 3. Claudin-5.tif]

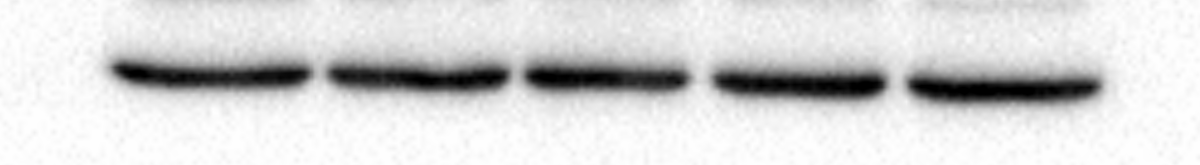

Supplement: Supplementary file 1 — Additional file 1. [file 12906_2022_3635_MOESM1_ESM.zip › actin(JAM-A).tif]

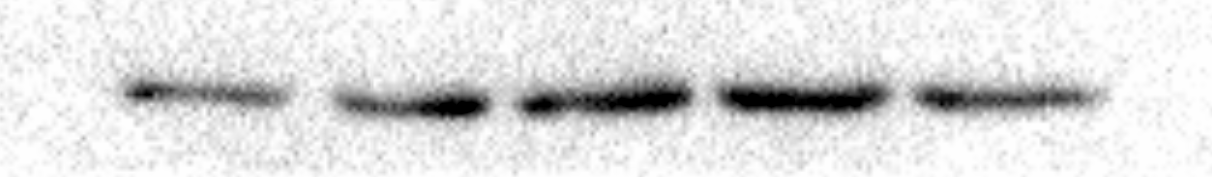

Supplement: Supplementary file 1 — Additional file 1. [file 12906_2022_3635_MOESM1_ESM.zip › actin(ZO-1).tif]

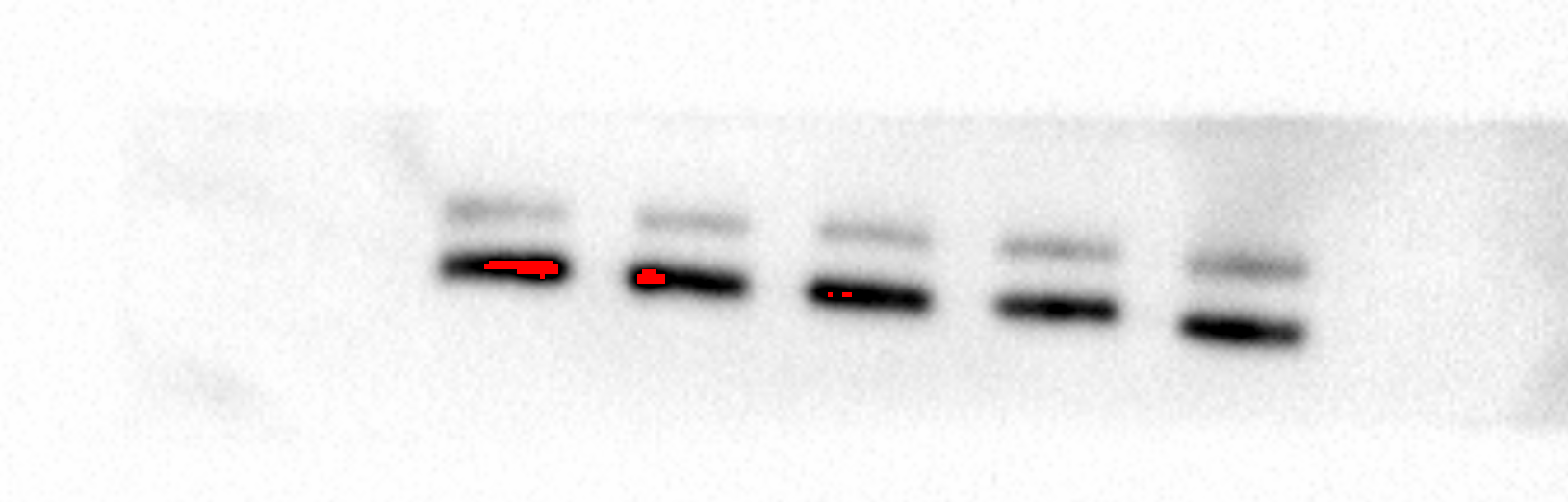

Supplement: Supplementary file 1 — Additional file 1. [file 12906_2022_3635_MOESM1_ESM.zip › Bcl-2 Bax_Exposure_11.8sec.tif]

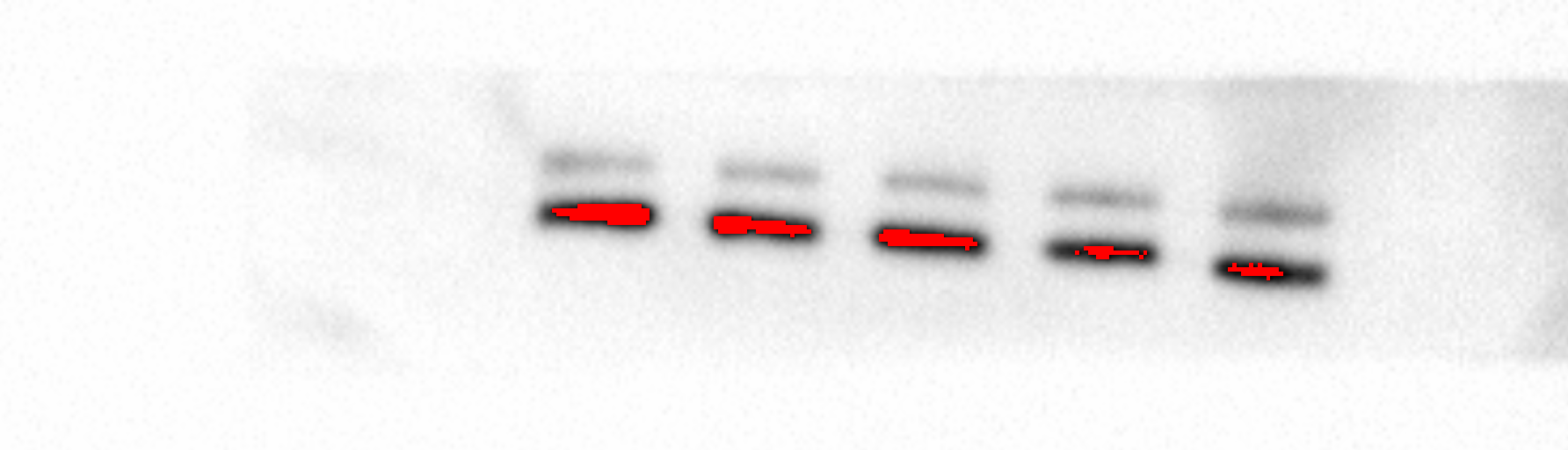

Supplement: Supplementary file 1 — Additional file 1. [file 12906_2022_3635_MOESM1_ESM.zip › Bcl-2 Bax_Exposure_15.4sec.tif]

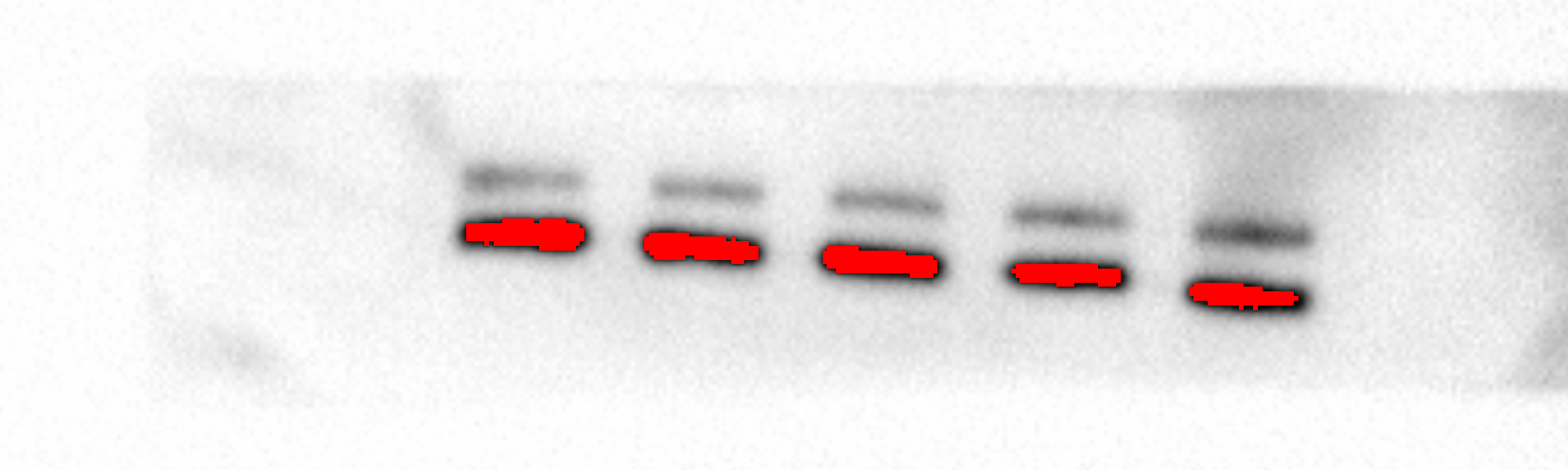

Supplement: Supplementary file 1 — Additional file 1. [file 12906_2022_3635_MOESM1_ESM.zip › Bcl-2 Bax_Exposure_22.6sec.tif]

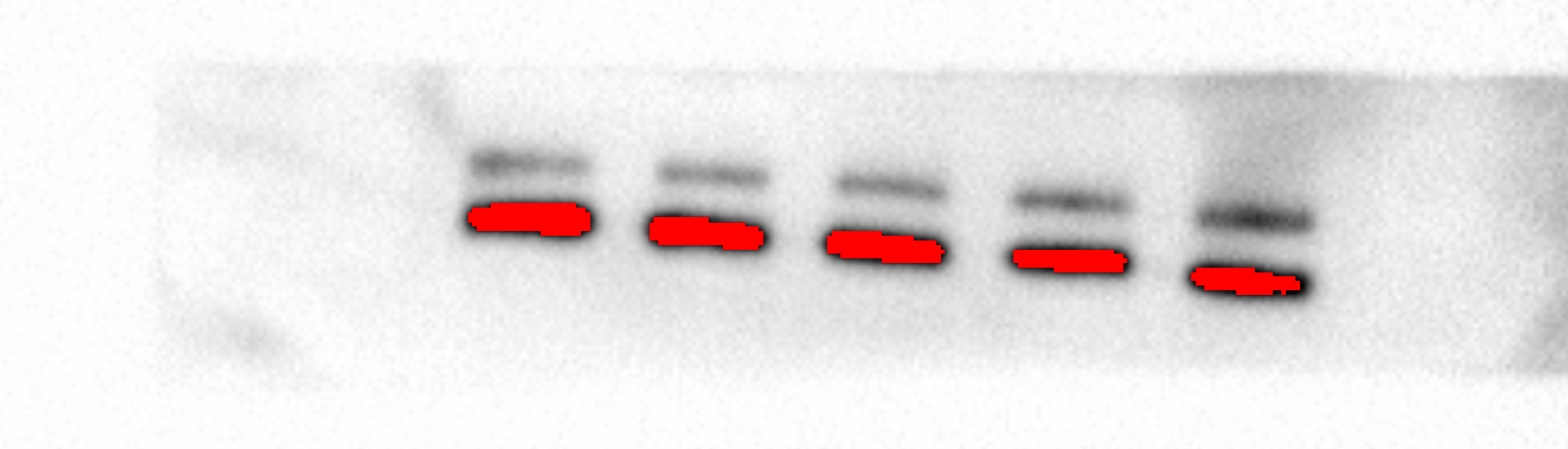

Supplement: Supplementary file 1 — Additional file 1. [file 12906_2022_3635_MOESM1_ESM.zip › Bcl-2 Bax_Exposure_25.0sec.tif]

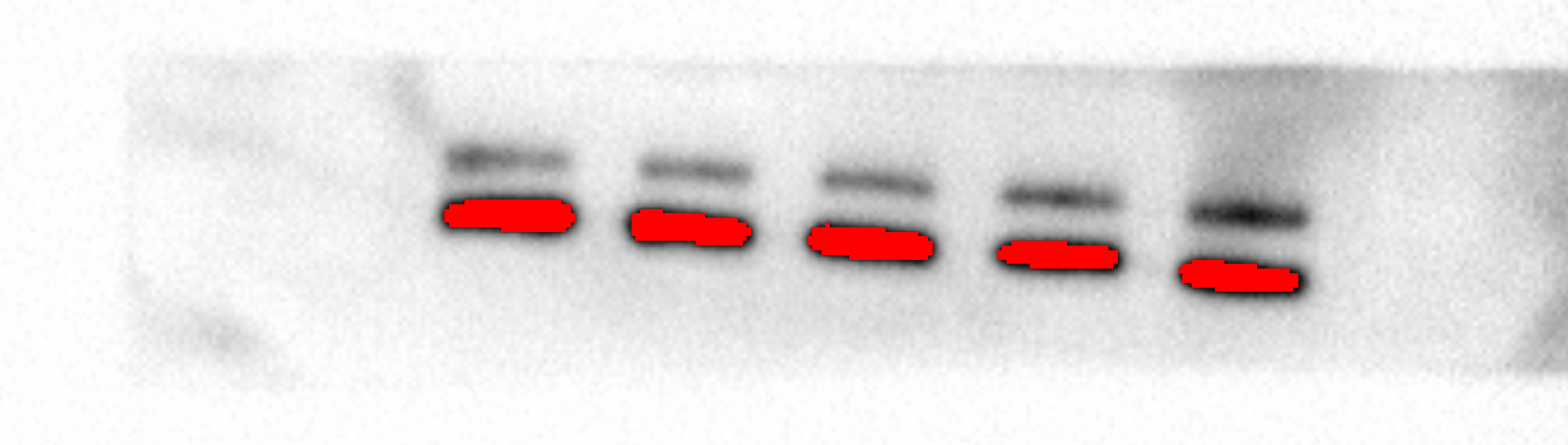

Supplement: Supplementary file 1 — Additional file 1. [file 12906_2022_3635_MOESM1_ESM.zip › Bcl-2 Bax_Exposure_29.8sec.tif]

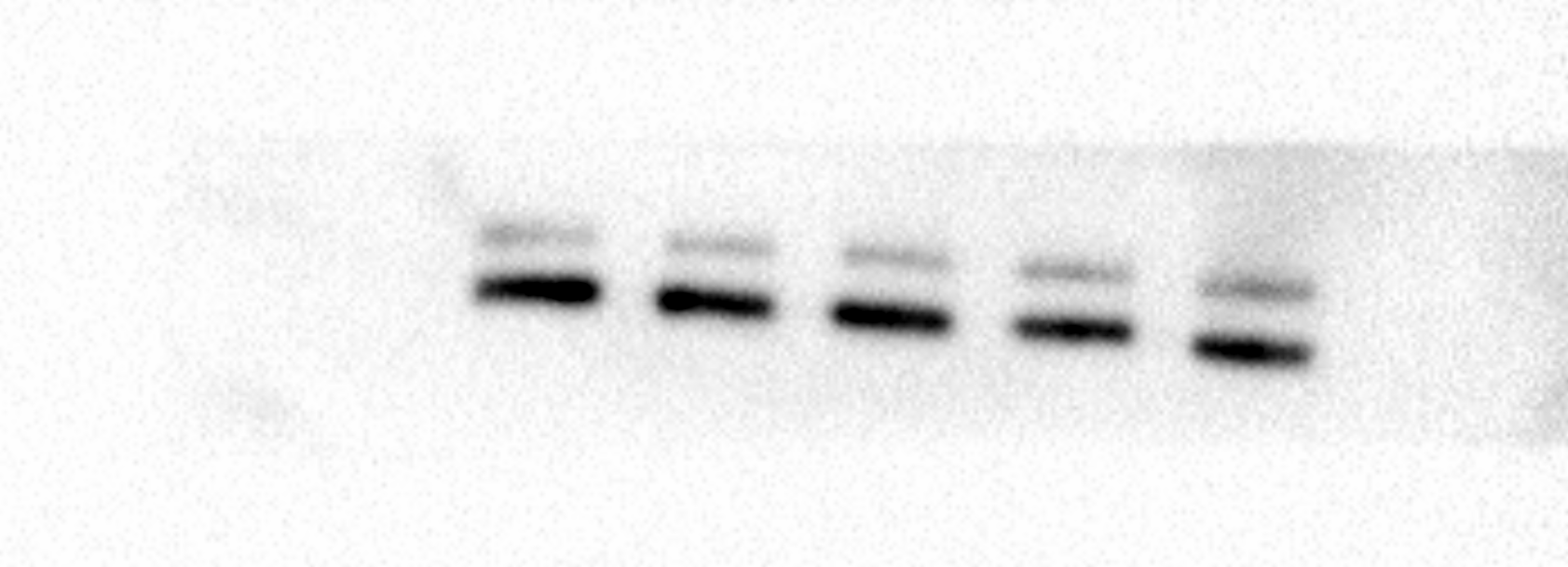

Supplement: Supplementary file 1 — Additional file 1. [file 12906_2022_3635_MOESM1_ESM.zip › Bcl-2 Bax_Exposure_4.6sec.tif]

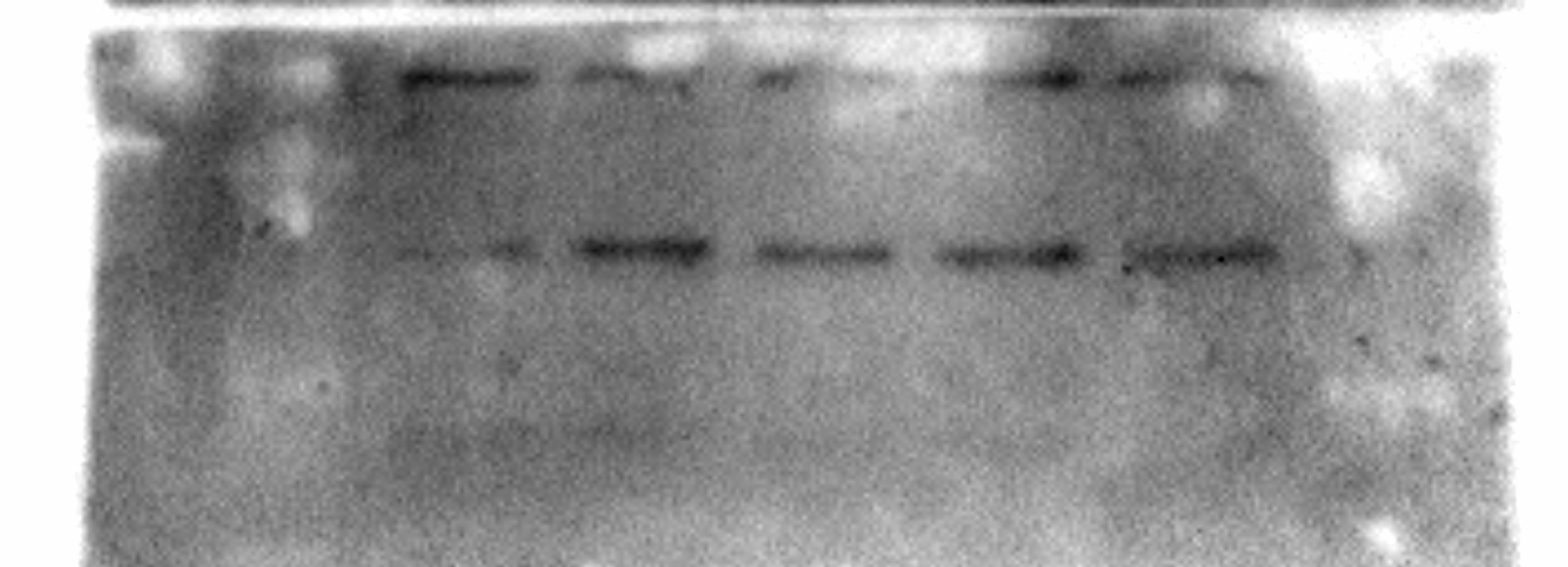

Supplement: Supplementary file 1 — Additional file 1. [file 12906_2022_3635_MOESM1_ESM.zip › Caspase-8 Cleaved caspase-8_Exposure_35.6sec.tif]

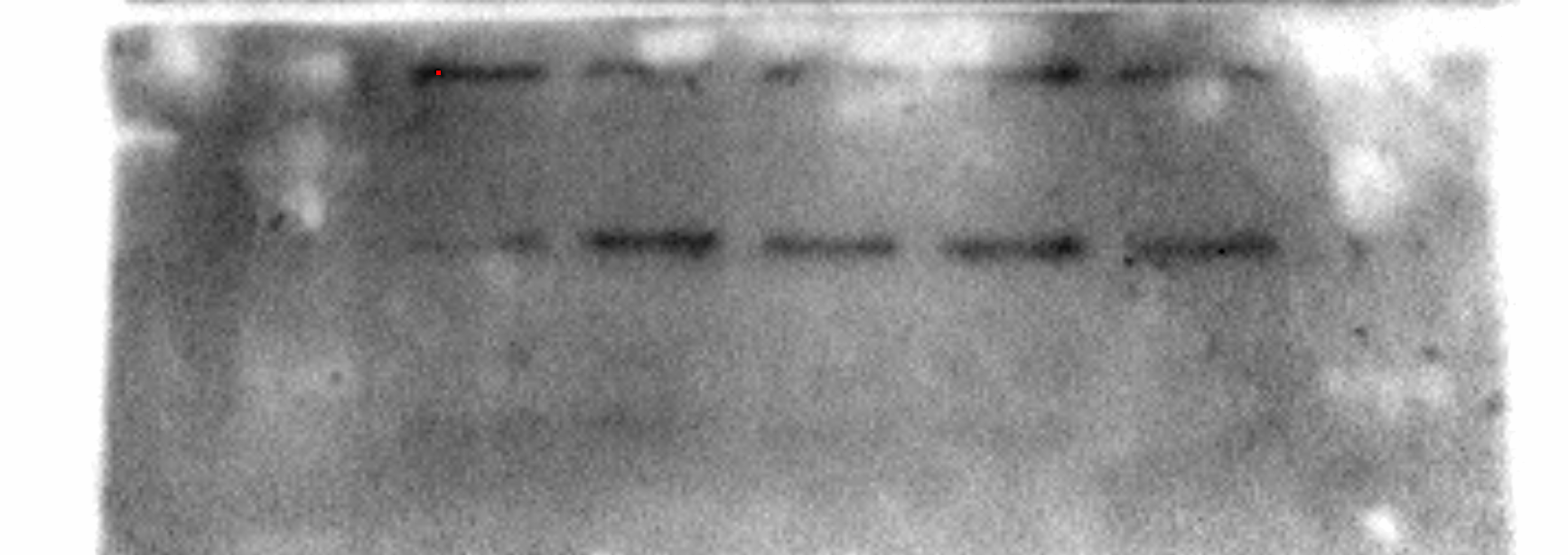

Supplement: Supplementary file 1 — Additional file 1. [file 12906_2022_3635_MOESM1_ESM.zip › Caspase-8 Cleaved caspase-8_Exposure_38.6sec.tif]

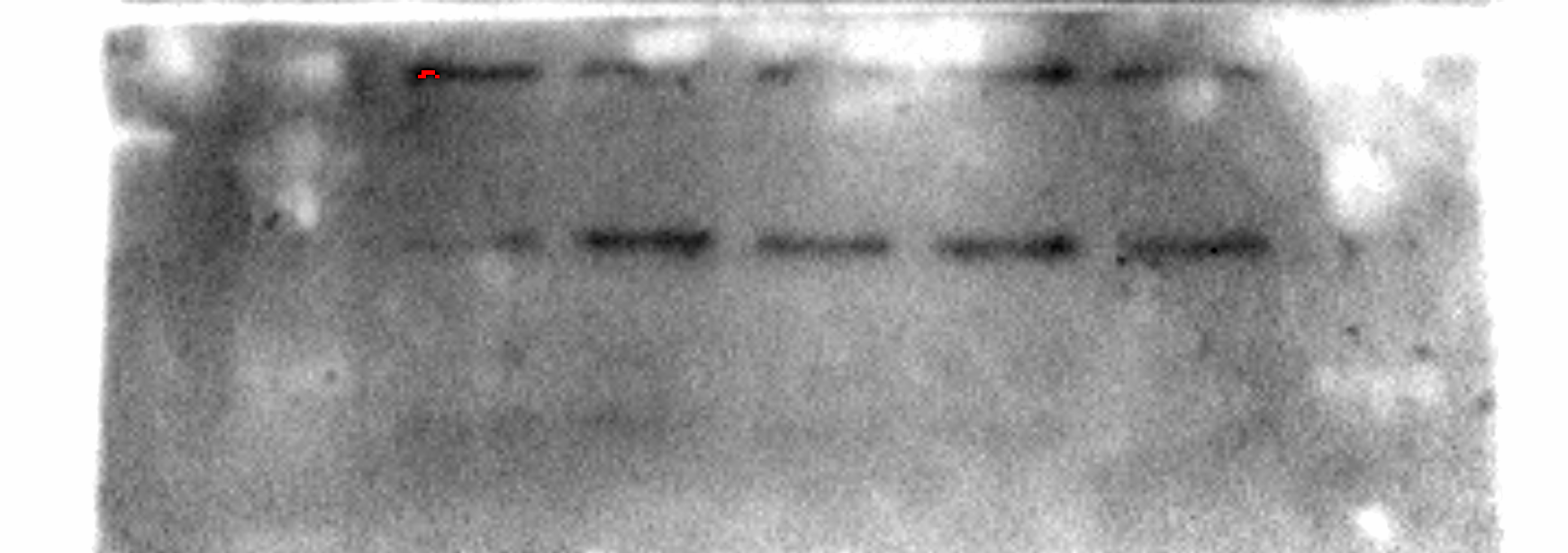

Supplement: Supplementary file 1 — Additional file 1. [file 12906_2022_3635_MOESM1_ESM.zip › Caspase-8 Cleaved caspase-8_Exposure_40.1sec.tif]

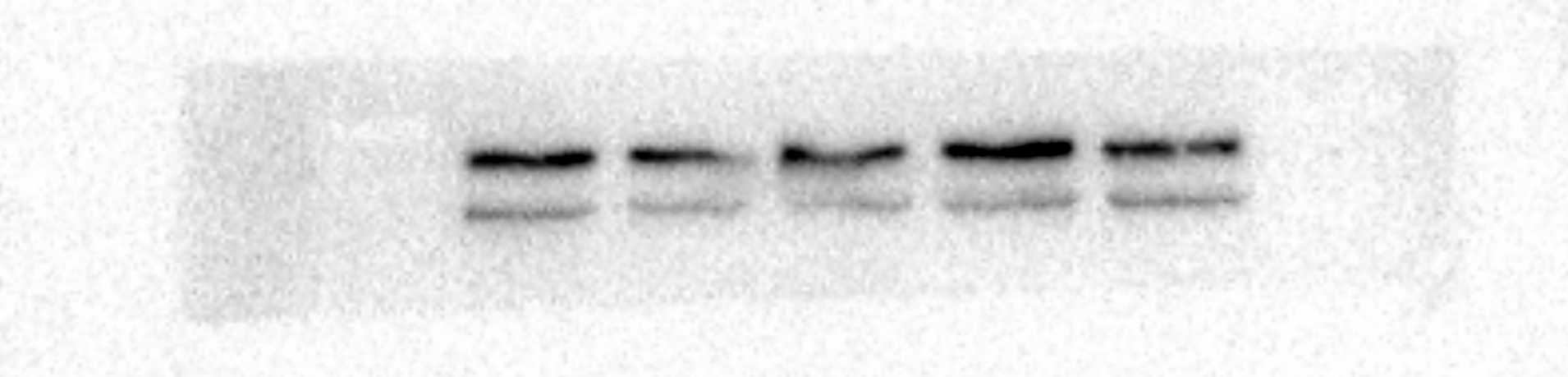

Supplement: Supplementary file 1 — Additional file 1. [file 12906_2022_3635_MOESM1_ESM.zip › Caspase-8 Cleaved caspase-8_Exposure_62.7sec.tif]

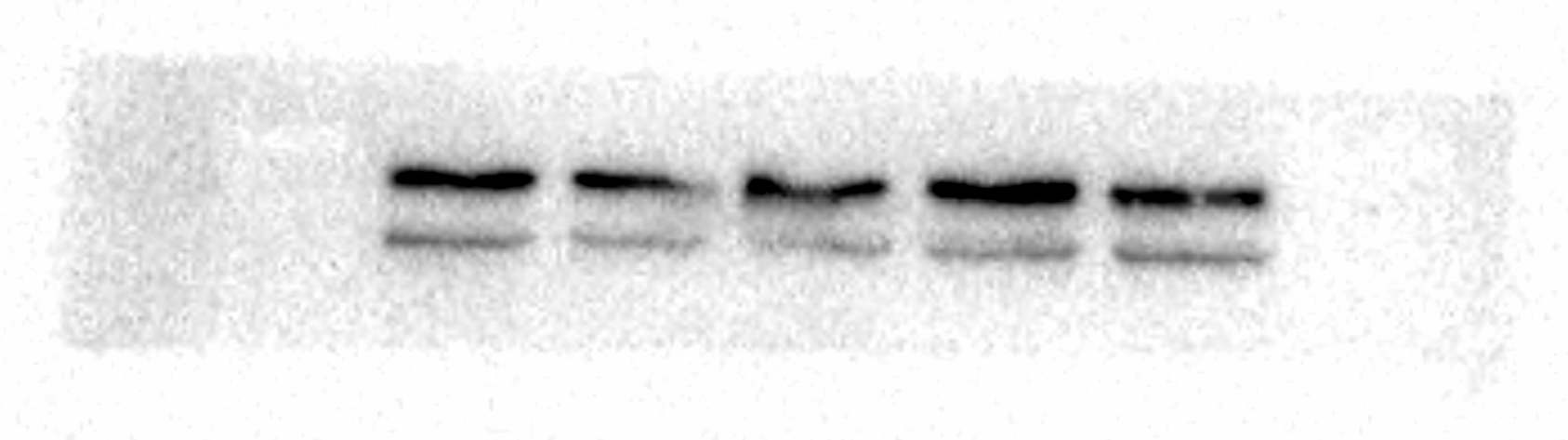

Supplement: Supplementary file 1 — Additional file 1. [file 12906_2022_3635_MOESM1_ESM.zip › Caspase-8 Cleaved caspase-8_Exposure_65.7sec.tif]

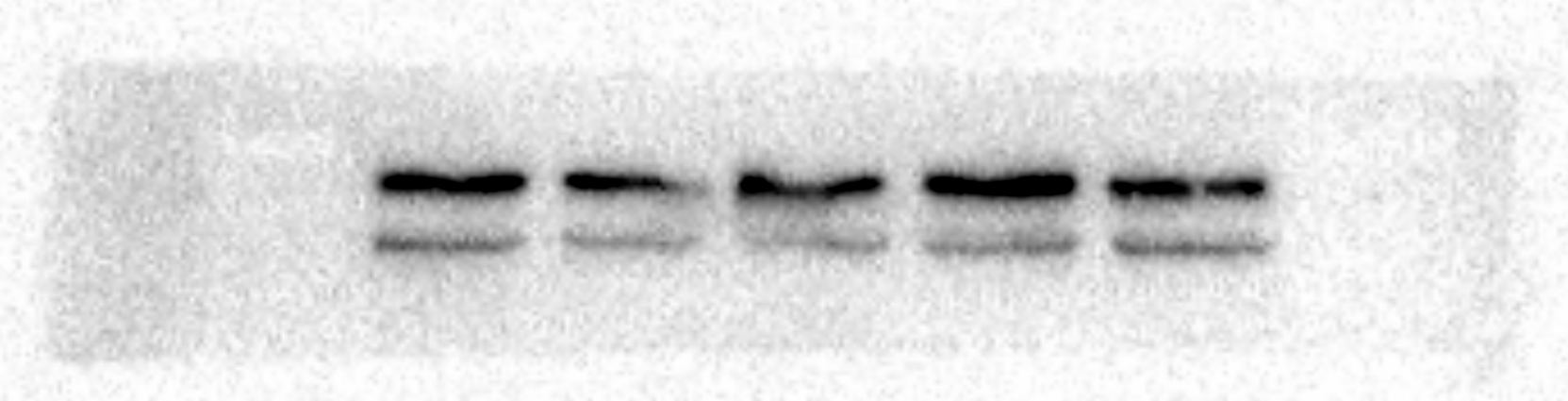

Supplement: Supplementary file 1 — Additional file 1. [file 12906_2022_3635_MOESM1_ESM.zip › Caspase-8 Cleaved caspase-8_Exposure_67.2sec.tif]

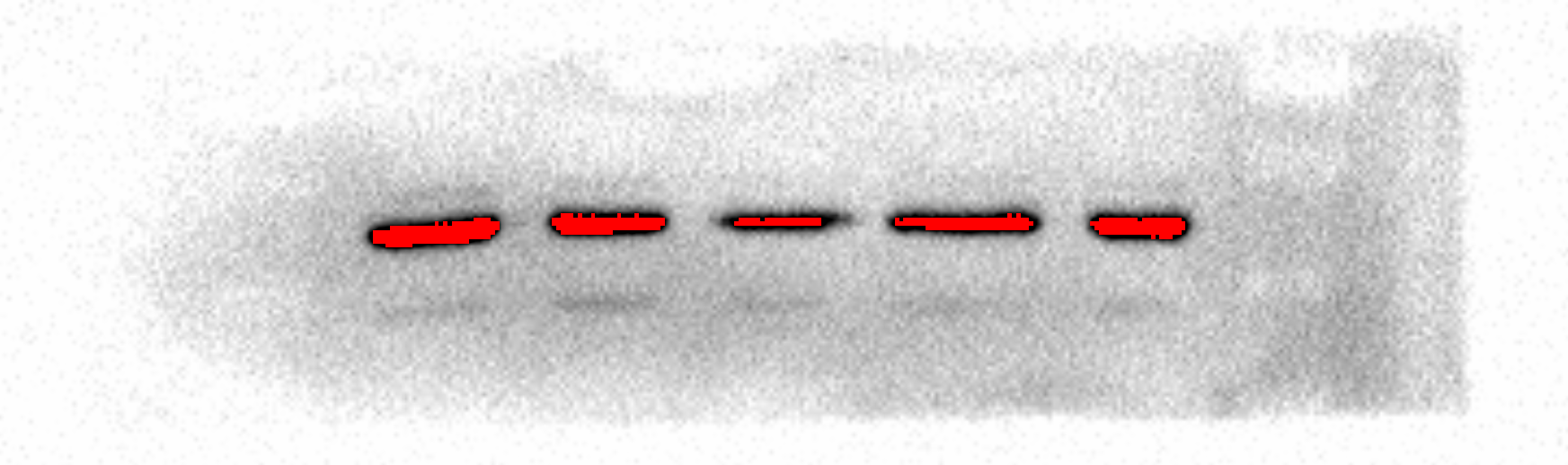

Supplement: Supplementary file 1 — Additional file 1. [file 12906_2022_3635_MOESM1_ESM.zip › Caspase-9 Cleaved caspase-9_Exposure_105.6sec-.tif]

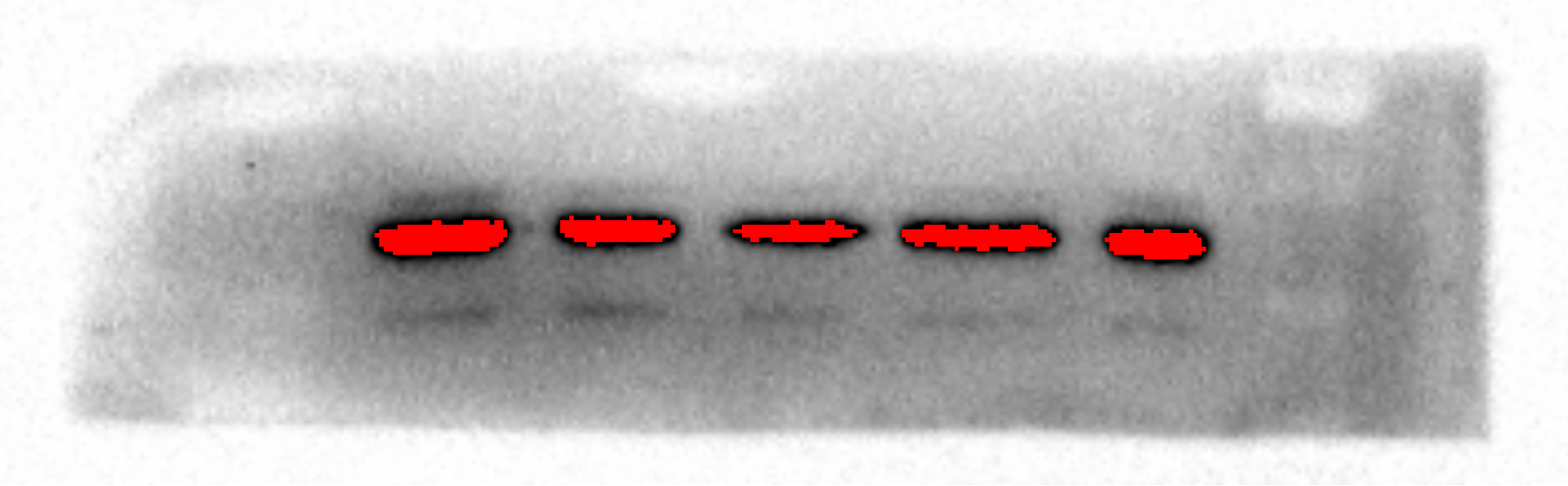

Supplement: Supplementary file 1 — Additional file 1. [file 12906_2022_3635_MOESM1_ESM.zip › Caspase-9 Cleaved caspase-9_Exposure_139.7sec-.tif]

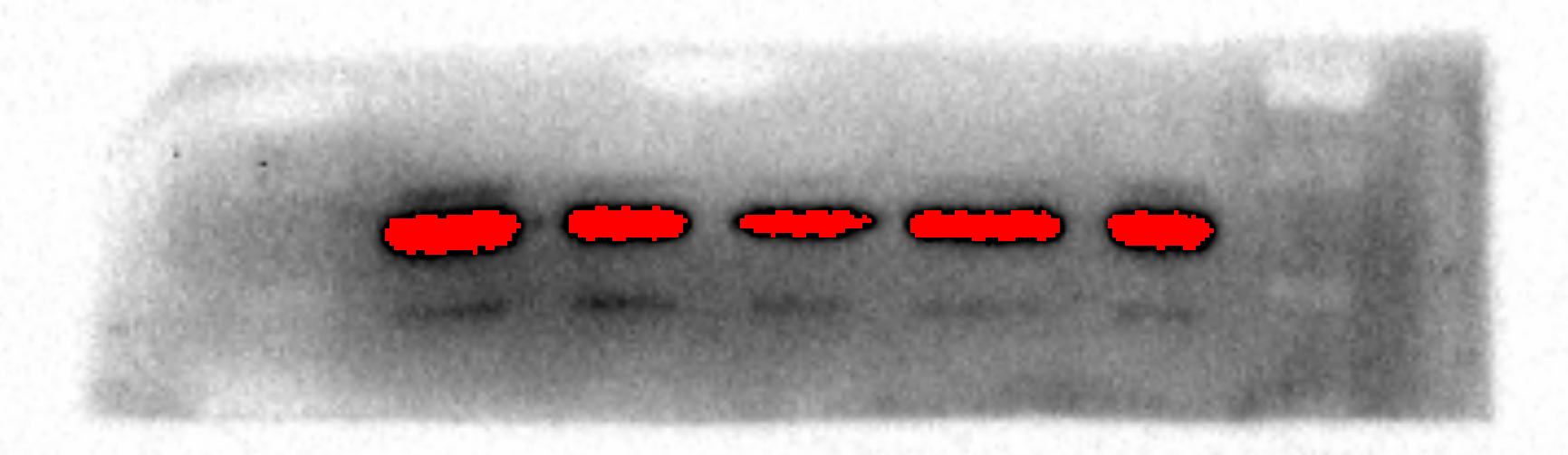

Supplement: Supplementary file 1 — Additional file 1. [file 12906_2022_3635_MOESM1_ESM.zip › Caspase-9 Cleaved caspase-9_Exposure_175.9sec-.tif]

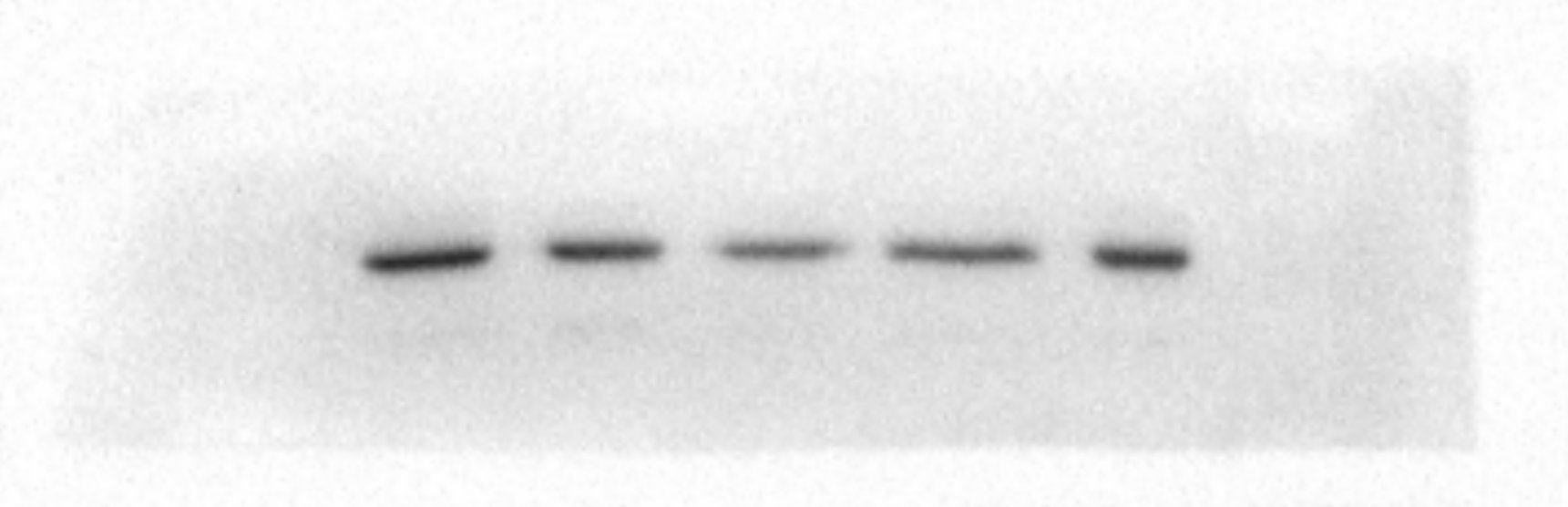

Supplement: Supplementary file 1 — Additional file 1. [file 12906_2022_3635_MOESM1_ESM.zip › Caspase-9 Cleaved caspase-9_Exposure_21.1sec.tif]

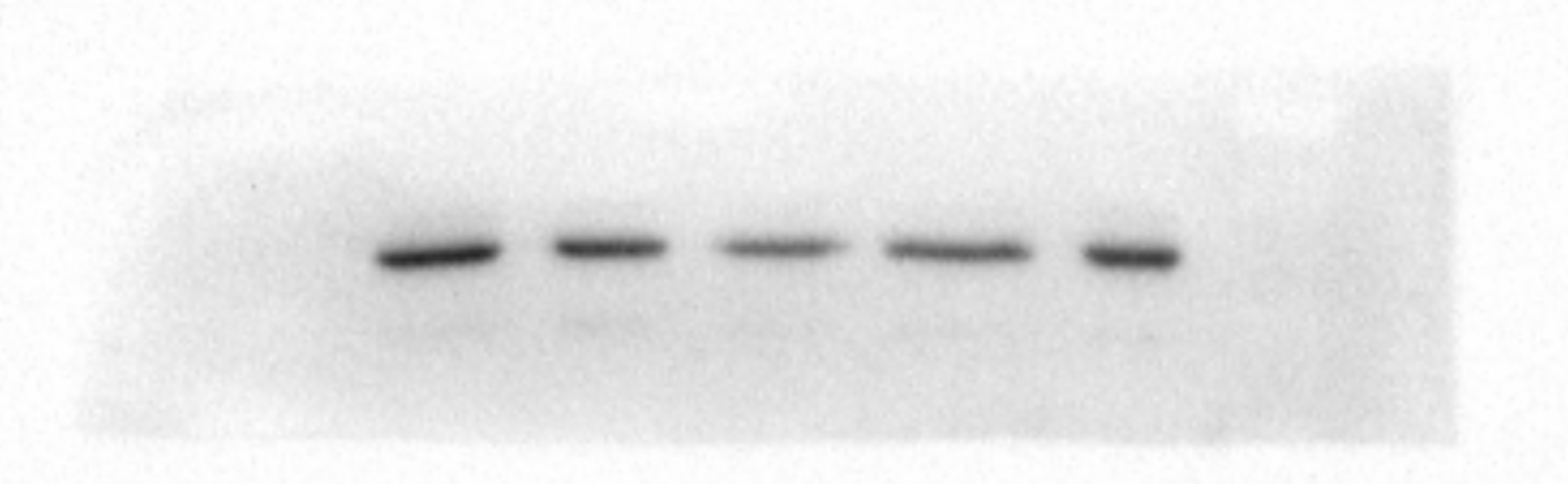

Supplement: Supplementary file 1 — Additional file 1. [file 12906_2022_3635_MOESM1_ESM.zip › Caspase-9 Cleaved caspase-9_Exposure_31.1sec.tif]

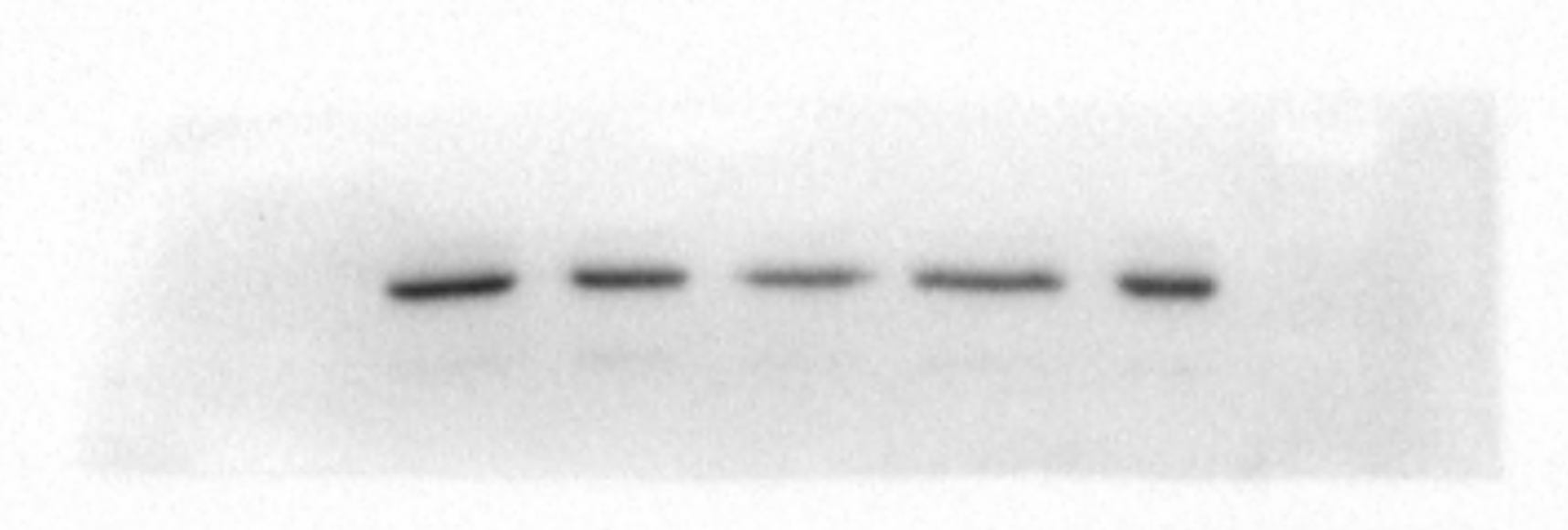

Supplement: Supplementary file 1 — Additional file 1. [file 12906_2022_3635_MOESM1_ESM.zip › Caspase-9 Cleaved caspase-9_Exposure_37.2sec.tif]

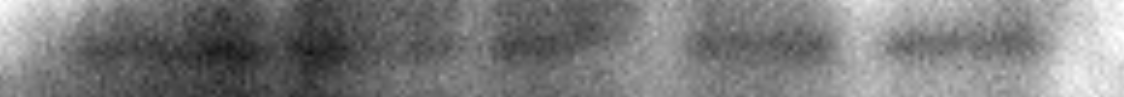

Supplement: Supplementary file 1 — Additional file 1. [file 12906_2022_3635_MOESM1_ESM.zip › JAM-A.tif]

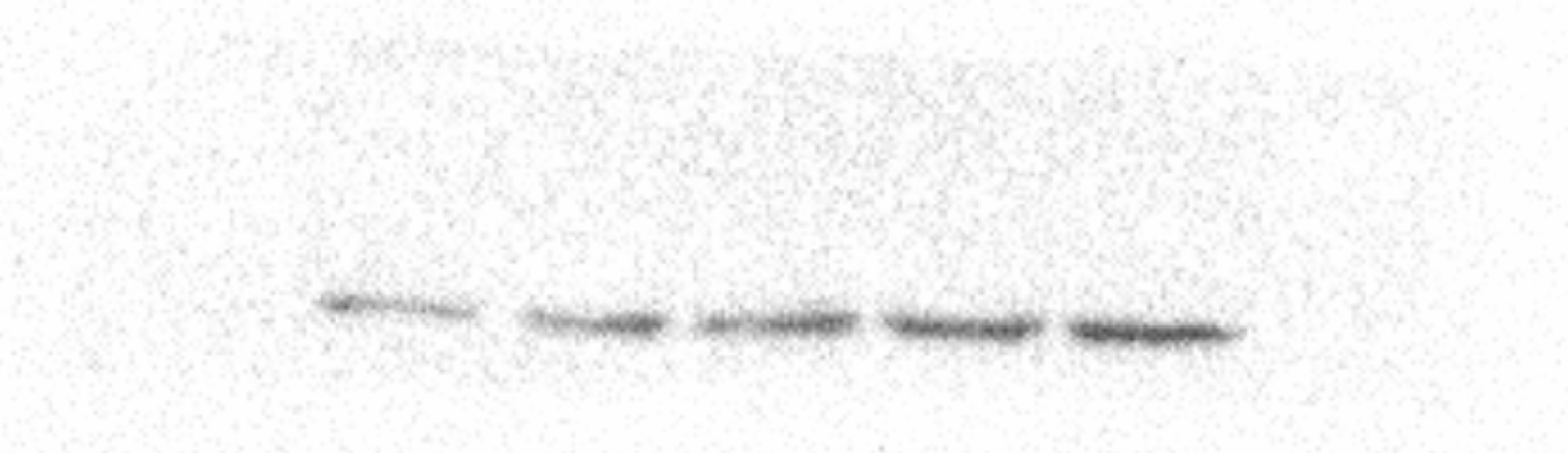

Supplement: Supplementary file 1 — Additional file 1. [file 12906_2022_3635_MOESM1_ESM.zip › Mfsd2a_Exposure_100.0sec.tif]

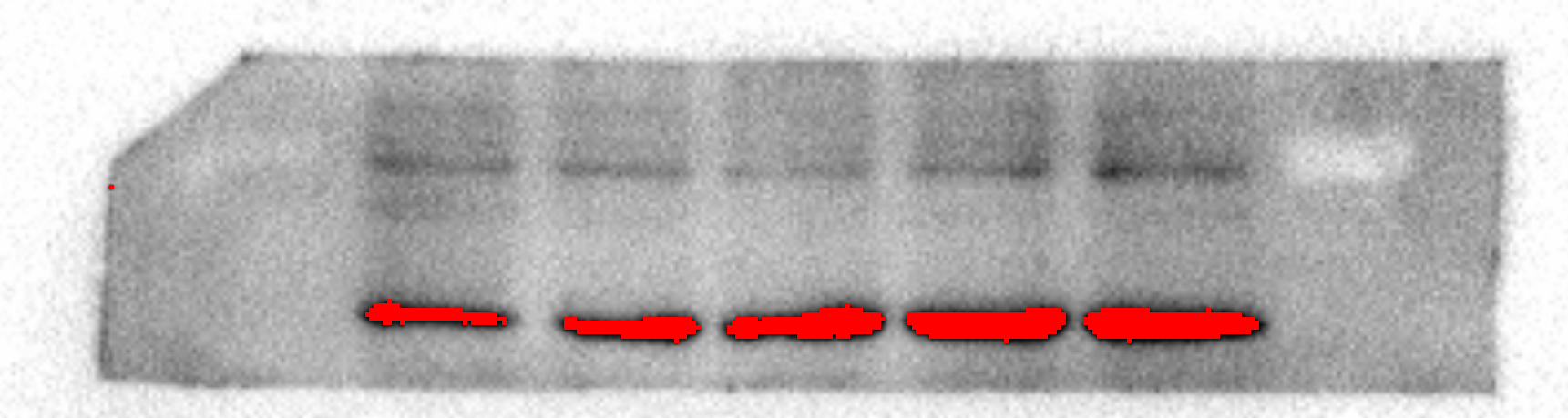

Supplement: Supplementary file 1 — Additional file 1. [file 12906_2022_3635_MOESM1_ESM.zip › Mfsd2a_Exposure_107.9sec.tif]

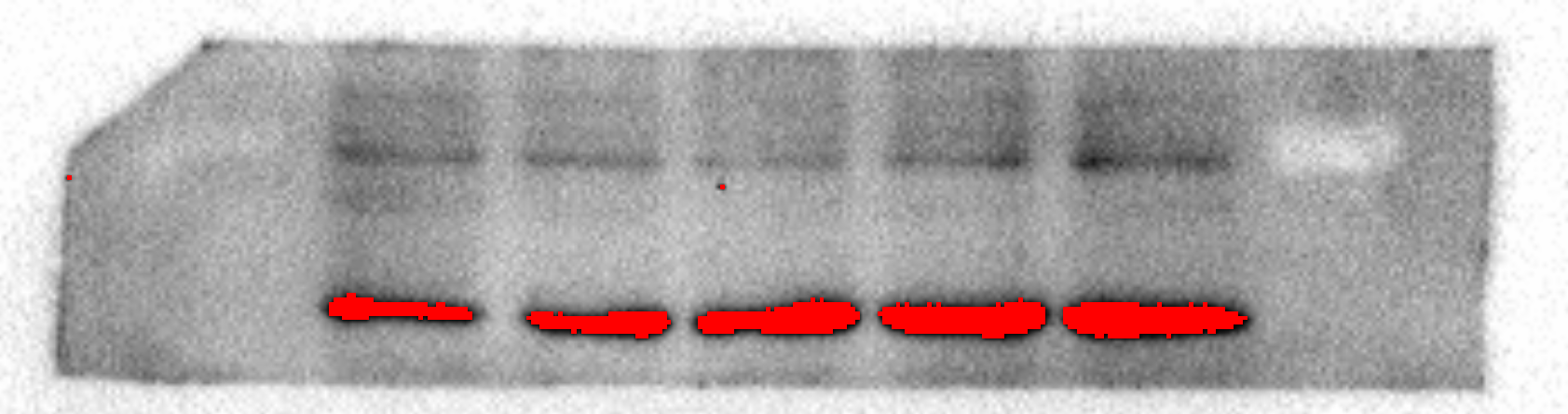

Supplement: Supplementary file 1 — Additional file 1. [file 12906_2022_3635_MOESM1_ESM.zip › Mfsd2a_Exposure_116.9sec.tif]

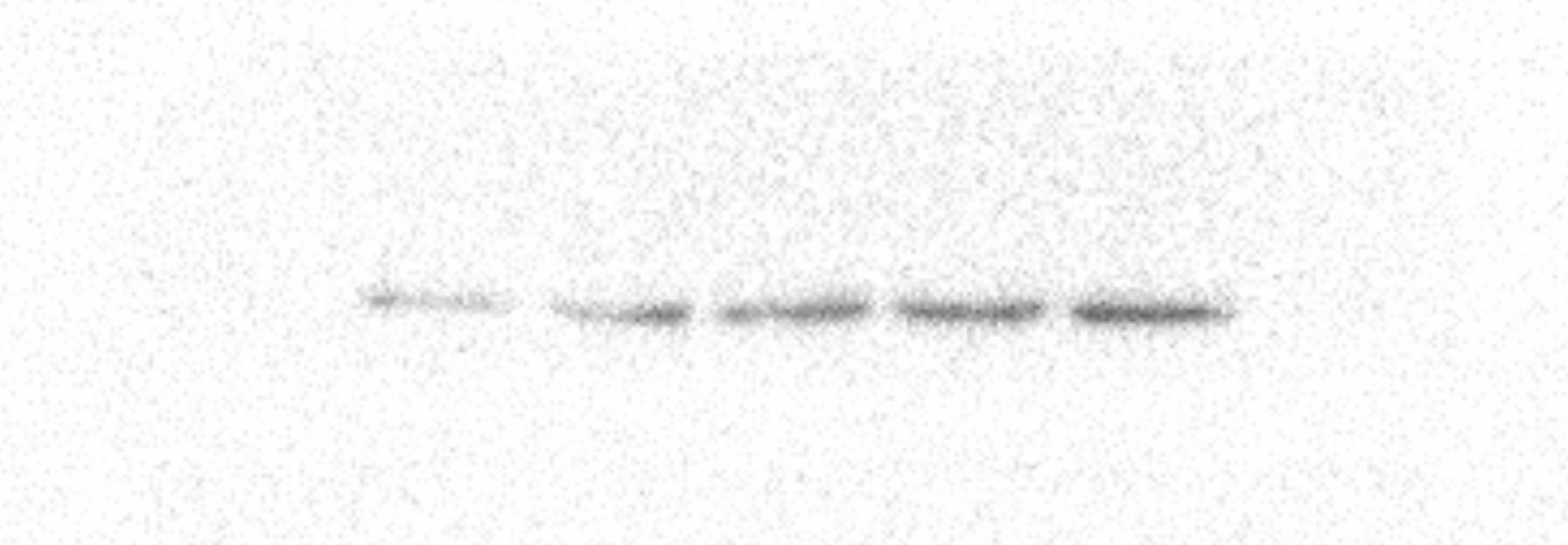

Supplement: Supplementary file 1 — Additional file 1. [file 12906_2022_3635_MOESM1_ESM.zip › Mfsd2a_Exposure_71.0sec.tif]

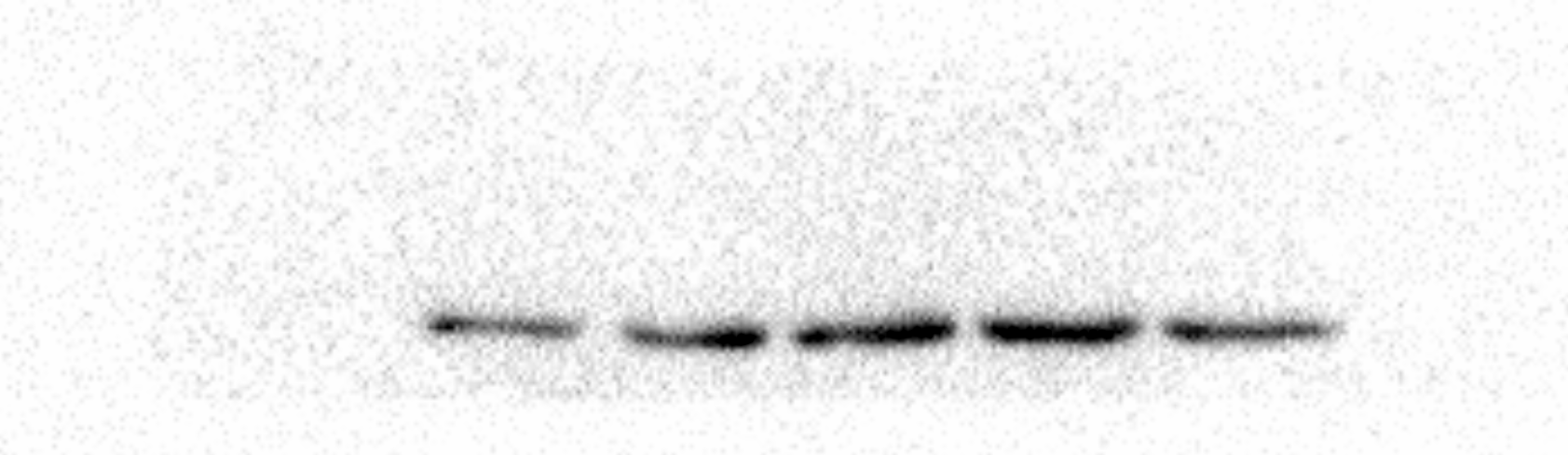

Supplement: Supplementary file 1 — Additional file 1. [file 12906_2022_3635_MOESM1_ESM.zip › Mfsd2a_Exposure_98.0sec.tif]

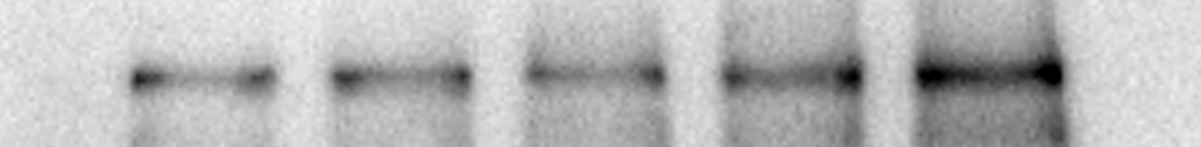

Supplement: Supplementary file 1 — Additional file 1. [file 12906_2022_3635_MOESM1_ESM.zip › ZO-1.tif]
